# Cohesin Is Required for Higher-Order Chromatin Conformation at the Imprinted *IGF2-H19* Locus

**DOI:** 10.1371/journal.pgen.1000739

**Published:** 2009-11-26

**Authors:** Raffaella Nativio, Kerstin S. Wendt, Yoko Ito, Joanna E. Huddleston, Santiago Uribe-Lewis, Kathryn Woodfine, Christel Krueger, Wolf Reik, Jan-Michael Peters, Adele Murrell

**Affiliations:** 1 Department of Oncology, University of Cambridge, Cancer Research UK Cambridge Research Institute, Cambridge, United Kingdom; 2 Institute of Molecular Pathology, Vienna, Austria; 3 Laboratory of Developmental Genetics and Imprinting, The Babraham Institute, Cambridge, United Kingdom; 4 Centre for Trophoblast Research, University of Cambridge, Cambridge, United Kingdom; Medical Research Council Human Genetics Unit, United Kingdom

## Abstract

Cohesin is a chromatin-associated protein complex that mediates sister chromatid cohesion by connecting replicated DNA molecules. Cohesin also has important roles in gene regulation, but the mechanistic basis of this function is poorly understood. In mammalian genomes, cohesin co-localizes with CCCTC binding factor (CTCF), a zinc finger protein implicated in multiple gene regulatory events. At the imprinted *IGF2-H19* locus, CTCF plays an important role in organizing allele-specific higher-order chromatin conformation and functions as an enhancer blocking transcriptional insulator. Here we have used chromosome conformation capture (3C) assays and RNAi–mediated depletion of cohesin to address whether cohesin affects higher order chromatin conformation at the *IGF2-H19* locus in human cells. Our data show that cohesin has a critical role in maintaining CTCF–mediated chromatin conformation at the locus and that disruption of this conformation coincides with changes in *IGF2* expression. We show that the cohesin-dependent, higher-order chromatin conformation of the locus exists in both G1 and G2 phases of the cell cycle and is therefore independent of cohesin's function in sister chromatid cohesion. We propose that cohesin can mediate interactions between DNA molecules in *cis* to insulate genes through the formation of chromatin loops, analogous to the cohesin mediated interaction with sister chromatids in *trans* to establish cohesion.

## Author Summary

Recent work has shown that cohesin, a protein best known for its role in holding sister chromatids together, and CTCF, a protein implicated in the formation of chromatin loops, localize to the same regions of DNA in mammalian genomes. This observation raised the intriguing possibility that cohesin might facilitate the role of CTCF in structuring chromatin. CTCF is well known for its role in regulating genomic imprinting at the *IGF2-H19* gene locus. Imprinted genes are widely studied due to their roles in fetal growth and cancer and have the unusual property of expressing only one parental copy of the gene. CTCF is thought to regulate imprinting of *IGF2* and *H19* by enabling DNA to form loops that separate the genes into silent or active domains. In this paper we describe, for the first time, the looping structure of the human *IGF2-H19* locus and show that cohesin stabilises CTCF–mediated DNA loops. Depletion of cohesin leads to disruption of long-range chromatin interactions and changes expression levels of the *IGF2* gene. This work adds a new level of understanding of how cohesin can play a role in gene expression.

## Introduction

Cohesin is an evolutionarily conserved protein complex composed of the core subunits, SMC1, SMC3, SCC1/RAD21 and SCC3/SA (reviewed in [1]). It has been proposed that cohesin mediates sister chromatid cohesion by embracing replicated DNA molecules as a ring [2]. Cohesin also has important roles in gene regulation in yeast, animals and humans (reviewed in [1],[3]). This regulatory function also exists during G1 phase and in post-mitotic cells, indicating that cohesin affects gene expression independent of its role in cohesion [4]–[6].

Cohesin mediates gene regulation at least in part by interaction with insulator elements [6]–[11]. Insulators are chromatin boundaries that separate gene promoters from regulatory elements. The only known protein that directly binds insulators in mammalian cells is the multi-functional zinc finger protein CTCF (CCCTC binding factor, reviewed in [12]). Several studies have recently identified co-localisation of CTCF and cohesin in mammalian genomes and have shown that CTCF is needed to recruit cohesin to these binding sites [6], [9]–[11]. Remarkably, although CTCF can associate with its binding sites in the absence of cohesin, its enhancer blocking activity depends on cohesin [6],[10]. It has, therefore, been speculated that CTCF may mediate transcriptional insulation by recruiting cohesin to particular sites in the genome [6], but it remains unknown how cohesin controls gene regulation at these sites.

The *IGF2-H19* locus plays a role in the aetiology of embryonic growth disorders and in various cancers (reviewed in [13]). A CTCF mediated insulator sequence plays a role in the reciprocal imprinting of *IGF2* and *H19* genes [14]–[16]. This insulator is located upstream of the *H19* gene and is known as the imprinting control region (ICR). It acquires methylation on the paternal allele during male germ cell development and is therefore also called the *H19* differentially methylated region (DMR) or domain (DMD). CTCF binding at the insulator prevents the *IGF2* gene from accessing enhancers downstream of the *H19* gene. This physical separation is thought to maintain the silence of the maternal *IGF2* allele. On the methylated paternal allele CTCF is excluded from binding and the *IGF2* promoters can interact with the enhancers [14],[15]. In mice it has been demonstrated that higher order chromatin conformation at this locus differs between the maternal and paternal alleles and that CTCF binding is essential for the formation of chromatin loops on the maternal allele [17]–[22]. In addition to the ICR and the downstream enhancers, several additional regulatory regions have been described surrounding the locus. At the 5′end of *IGF2* there is a differentially methylated region, DMR0, which in human has variable methylation in somatic tissues and is hypomethylated in cancers [23]–[25]. In the intervening sequence between *IGF2* and *H19* there is a Centrally Conserved DNase I hypersensitive domain (CCD), which in mice has tissue specific enhancer functions [26]–[30]. In humans the functions of the CCD and DMR0 are unknown.

**Figure 1:**
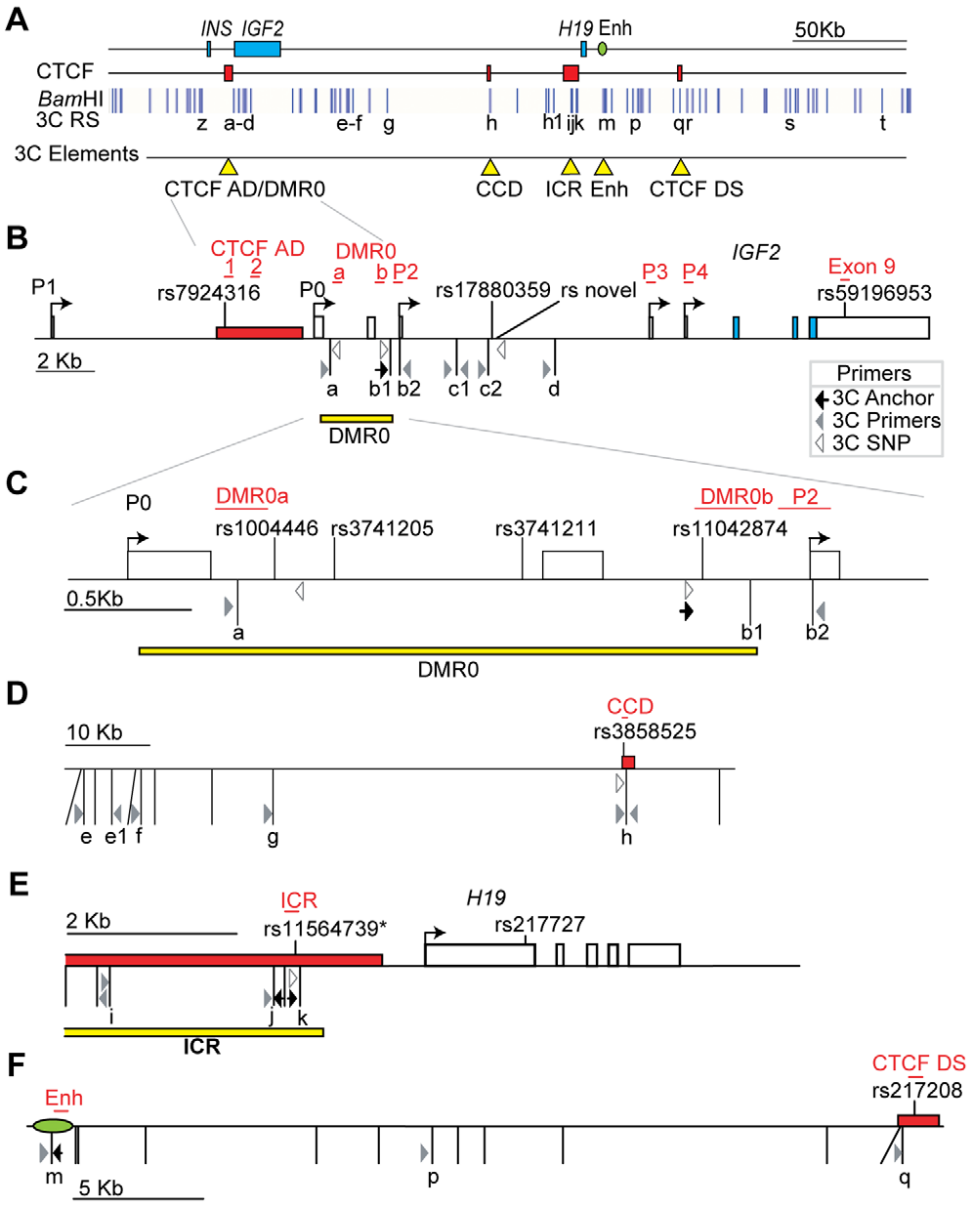
Schematic representation of the *IGF2-H19* locus. (A) The Insulin (*INS*), Insulin-like Growth Factor 2 (*IGF2*), and *H19* genes (blue boxes) are displayed together with the downstream enhancer (Enh; green oval). CTCF/cohesin binding regions are indicated by red rectangles, *Bam*HI restriction sites by vertical blue lines, and 3C restriction sites (3C RS) by letters. The 3C elements (yellow triangles) represent regions analysed by 3C; the *IGF2* upstream region (CTCF AD/DMR0), the Centrally Conserved DNase I hypersensitive domain (CCD), the Imprinting Control Region (ICR), the *H19* downstream enhancers, and a CTCF binding Downstream Site (CTCF DS). (B–F) Enlargements of the 3C elements and surrounding genomic areas indicating primer positions relative to restriction sites. Anchor primers are indicated with dark arrows, reciprocal 3C primers with grey arrowheads, and primers used for SNP analysis with open arrowheads. (B) Enlarged region of the *IGF2* gene. The *IGF2* upstream region is composed of the CTCF region adjacent to DMR0 (CTCF AD; red box) and the DMR0 (yellow bar). Promoters (P1 to P4), exons (empty boxes for non-coding and blue boxes for coding), and single nucleotide polymorphisms (SNP; rs numbers) are indicated. Red type and underlying bars represent ChIP amplicons. The closest *Bam*HI restriction site to the CTCF AD is restriction site a. Restriction site b1 is within DMR0, but is also close to the P2 promoter. Restriction sites b2, c1, c2, and d are between the P2 and P3 promoters. (C) Detail of the DMR0 region to indicate SNP positions in the a-b1 restriction fragment. (D) The intervening region between *IGF2* and *H19* showing the position of the CCD relative to restriction sites e to h. (E) Enlargement of the ICR (yellow bar) and *H19* gene (open boxes). Restriction sites i to k lie in the CTCF binding regions (red box) and ICR rs115647398* is a SNP in HB2 that is 2 bp away from the annotated rs115647398. (F) Detail of the enhancers region and CTCF DS. Primer information is available in Table S2 and Table S3.

Because CTCF is needed for the recruitment of cohesin to insulator sequences, we speculated that cohesin, with its unique capability of holding DNA strands together, is required for the formation and stabilisation of CTCF-dependent chromatin loops. To test this hypothesis we undertook quantitative Chromatin Conformation Capture (q3C) analysis [31] of the human *IGF2-H19* locus (Figure 1) and examined CTCF-dependent chromatin loops after cohesin depletion by RNAi. Our results indicate that cohesin has an important role in long-range interactions between CTCF sites, whereas CTCF independent chromatin associations do not require the presence of cohesin. Cohesin may, therefore, contribute to gene regulation at CTCF sites by mediating the formation of chromatin loops.

## Results

**Figure 2:**
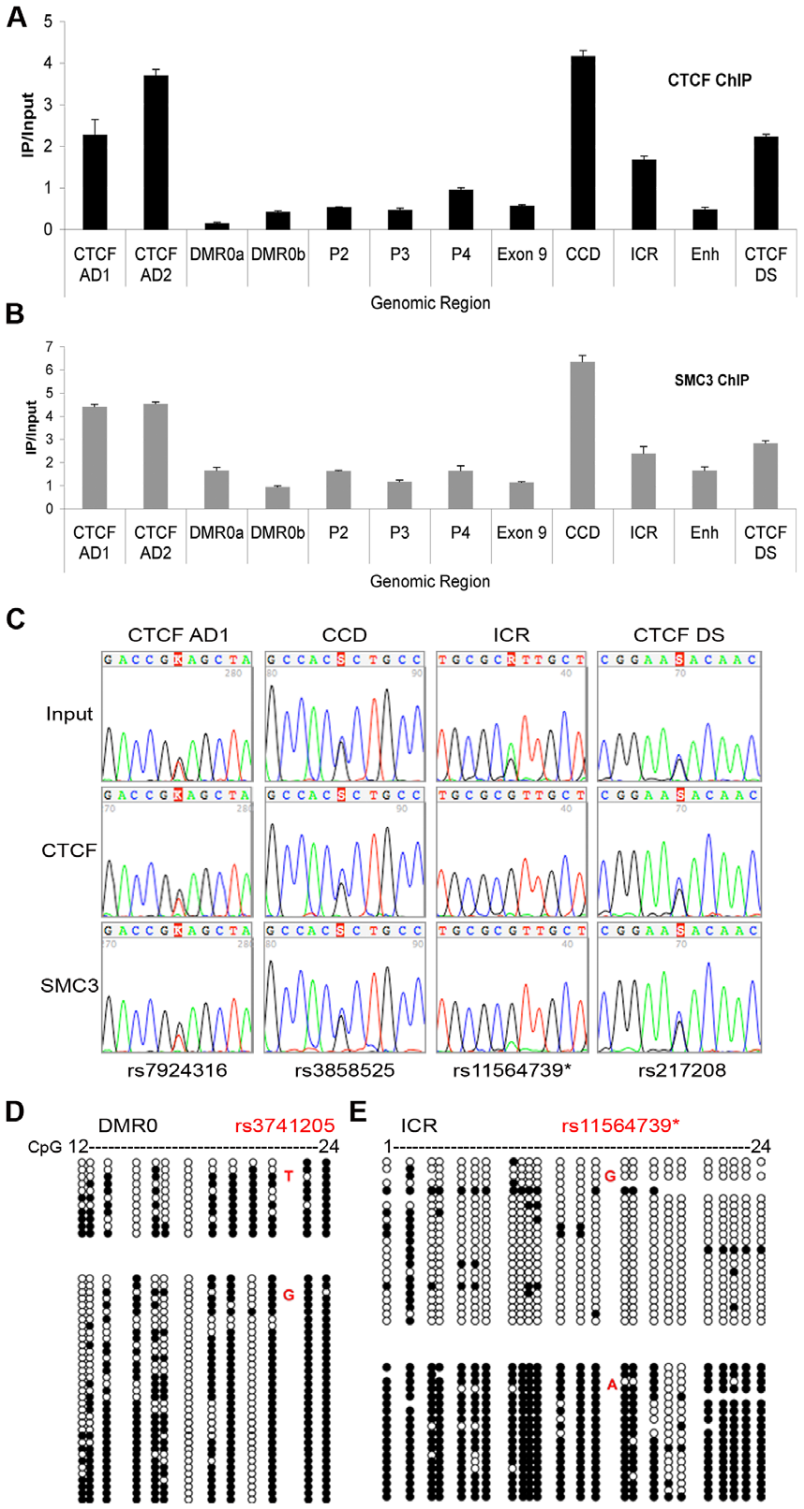
CTCF and the cohesin binding at *IGF2-H19* region in HB2 cells. (A) Enrichment of PCR products from CTCF bound regions in the HB2 cell line over the Input (IP/Input). (B) Enrichment (IP/Input) of cohesin at same regions. Strong enrichment for CTCF and cohesin is seen at the CTCF sites adjacent to DMR0 (CTCF AD1 and 2), the CCD and a CTCF downstream of *H19* (CTCF DS). Error bars represent standard deviations of 3 replicate PCRs. (C) SNP analysis of CTCF and cohesin ChIP for the CTCF sites adjacent to DMR0, (CTCF AD1), the CCD, ICR, and the CTCF DS. Enriched sequences for CTCF and cohesin binding are biallelic for the CTCF site adjacent to DMR0 and to the CCD at the given SNPs. At the ICR, the G-allele at rs11564739* was preferentially enriched after CTCF and cohesin ChIP. (D) Methylation status of the DMR0 and (E) the ICR. Each horizontal group of circles represents a single sequence. Specific alleles identified by the SNPs shown in red are grouped. Each circle represents a single CpG. Gaps between circles indicate that the CpGs are not adjacent. Open circles represent unmethylated CpGs, filled circles represent methylated CpGs. The allele that binds CTCF at the ICR is the unmethylated G-allele.

We chose to study the role of cohesin at the *IGF2-H19* locus in human cells because we are interested in the effects of chromatin looping in human cells. We have previously developed protocols for depletion of cohesin subunits by RNAi for human cells [6]. We used a diploid human breast epithelial cell line, HB2, from which cohesin could be depleted, which could be synchronised in the cell cycle, and which contained informative Single Nucleotide Polymorphisms (SNPs), enabling us to distinguish between alleles at the *IGF2-H19* locus. *H19* expression in these cells is monoallelic and as with most normal adult tissues *IGF2* is expressed only at basal levels [32],[33]. Bisulphite sequencing confirmed that this cell line has monoallellic methylation at the *H19* ICR (Figure 2E), indicating correct imprinting at this locus. The ICR acquires its methylation in the male germ line and maintains it in all normal tissues even if *IGF2* is tissue specifically or developmentally down-regulated [34].

### Co-localisation of CTCF and cohesin within the *IGF2-H19* locus

To predict higher order interactions that may be directly mediated by CTCF and cohesin at this locus in HB2 cells, we performed locus-wide ChIP experiments with antibodies specific for CTCF and SMC3 (this subunit of cohesin was analysed due to the availability of validated ChIP grade antibodies [6]). We found that CTCF and cohesin co-localise at the ICR, at CTCF sites immediately adjacent to the DMR0, at CTCF sites in the CCD region [26],[27],[30] and also with CTCF sites downstream of the enhancers (Figure 2A and 2B), consistent with previous data from other cell lines [6],[35]. We refer to the CTCF sites adjacent to the DMR0 as “CTCF AD” and to those downstream of the enhancers as “CTCF DS” (Figure 1). In contrast to the above mentioned sites, CTCF and cohesin were not enriched at the DMR0, *IGF2* promoters or the enhancers downstream of *H19* (Figure 2A and 2B). SNP sequencing of ChIP-PCR samples indicated that CTCF and cohesin bind at all sites to both alleles, with exception of the ICR, where monoallelic binding was observed on the unmethylated allele (Figure 2C and 2E). The CTCF sites adjacent to the DMR0 and the CCD are not CpG rich and were unmethylated (data not shown).

### Locus-wide chromatin conformation studies at *IGF2-H19*

We determined the chromatin conformation at the human *IGF2-H19* locus through extensive q3C analyses with a *Bam*HI restriction enzyme, using primers within the ICR, enhancer, and two other CTCF sites (CTCF AD/DMR0 and CTCF DS) flanking the locus as anchors. *Bam*HI cuts the locus frequently, but there are no restriction sites within the CTCF AD. The nearest site to the CTCF AD is on the edge of the DMR0 region (restriction site a). For this reason we have regarded CTCF AD/DMR0 as a single 3C element in our analysis. The resulting data represent an average of association frequencies on both parental alleles across the whole locus. Random ligations are expected to decrease exponentially the further a restriction site is away from the anchor, while specific associations occur as “spikes” above the random ligation curve [36]. However, because the resolution of 3C is limited, multiple adjacent restriction sites within a 5 Kb stretch of DNA will associate with similar frequencies with a distant restriction site used as an anchor. The 3C signals therefore indicate proximities to interactions, rather than pinpoint the exact sequences involved in the interactions.

**Figure 3:**
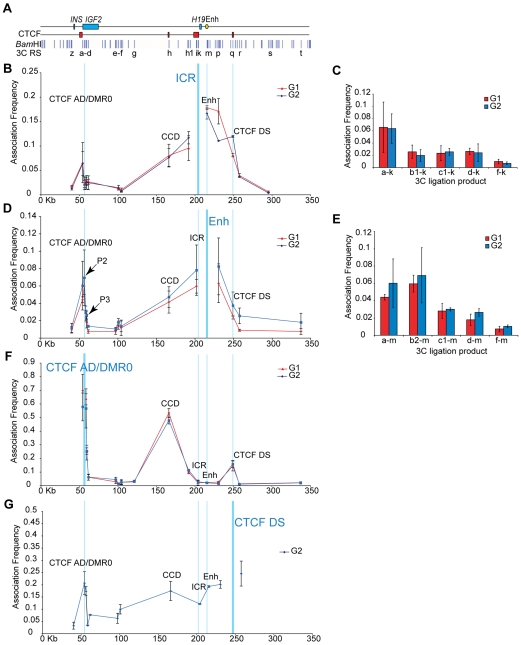
Chromatin conformation of the *IGF2-H19* locus is conserved between G1 and G2 phase of the cell cycle. (A) Schematic representation of a 350 Kb genomic region including *INS* and the *IGF2-H19* locus. Blue boxes represent the position of genes and the green oval depicts the localisation of the enhancer (Enh) (see also Figure 1). Similarly, red boxes in the second line represent the CTCF/cohesin binding regions CTCF AD, CCD, ICR, and CTCF DS. Vertical blue lines indicate the position of *Bam*HI restriction sites within the locus and letters point out the restriction sites analysed by 3C (3C RS) in the graphs and histograms shown in (B, D, F, G). The blue vertical lines in (B, D, F, G) link the positions of the restriction sites used as anchors in the different panels with the overview of the locus in (A). Thicker vertical lines indicate the position of the anchor in the respective graph. The anchor restriction sites fragments are: b1 in the CTCF AD/DMR0 region, k in the ICR, m in the enhancer and q in the CTCF DS region. The X-axis is labelled according to genomic position and position 0 was arbitrarily fixed 42 Kb upstream of *INS*. (B) Associations detected with the anchor site in the ICR (primer k). An association is present between the ICR and the CTCF AD/DMR0 region in both G1 and G2 phase and with the CTCF DS preferentially in G2. (C) The peak of association frequencies between the ICR and CTCF AD/DMR0 compared to random ligation (f–k) is shown in the histogram. Restriction site a is adjacent to the CTCF AD region (Figure 1) and the high association frequency between a and k might reflect directly the CTCF/cohesin binding to the CTCF AD. (D) Associations detected with an anchor in the enhancer (primer m). Peaks indicating interactions between the enhancer and the CTCF AD/DMR0 region can be seen in G1 and G2 phase cells. Positions of primers P2 and P3 are indicated. (E) The peak of association frequencies between the enhancer and CTCF AD/DMR0 region is shown in the histogram. Association values at restriction sites a–m report on the CTCF AD/DMR0 interaction with the enhancer, while b2-m, c1-m and d–m report on the *IGF2* promoters P2 and P3. (F) Associations with the CTCF AD/DMR0 region (restriction site b1 as anchor site). Very high association frequencies are visible for the interactions with the CCD and the distant CTCF DS in G1 and G2 phase cells, presumably due to the strong and biallelic binding of CTCF and cohesin at these sites. (G) Associations of the CTCF DS region (q anchor) throughout the locus during G2 phase. Reciprocal association peaks with the CTCF AD/DMR0 and the CCD are visible.

When a primer within the ICR was used as an anchor (primer k), weak but specific associations were detected with the CTCF AD/DMR0 region (restriction site a, Figure 3B and 3C). When the enhancer was used as an anchor (primer m) we were able to detect associations with a restriction site within the CTCF AD/DMR0 (restriction site a) as well as at restriction sites near the P2 and P3 promoters (sites b2, c1 and d, Figure 3D and 3E), despite low levels of *IGF2* expression in these cells. However, when an anchor primer was placed within the CTCF AD/DMR0 region (primer b1), we found strong association frequencies with the CCD (restriction site h) and the CTCF DS (restriction site q), but not with the ICR or the enhancer (Figure 3F). Because the DMR0 does not bind CTCF, it is possible that the associations between the CTCF AD/DMR0 and the CCD are caused by CTCF binding at the CTCF AD. To confirm that the CTCF AD/DMR0 region interacts with the distant CTCF DS sites we also used a primer within the CTCF DS (primer q) as an anchor. As expected, this revealed associations between CTCF DS and the CTCF AD/DMR0 region (restriction sites a and b1), and in addition showed associations with the CCD (restriction site h) (Figure 3G). Locus-wide q3C analysis was also performed using *Bgl*II as a restriction enzyme which cuts in the CTCF AD site. These experiments revealed interactions between the CTCF AD anchor, the CCD and CTCF DS as well as ICR anchor and the CTCF AD (Figure S1 and Figure S2), similar to what we had obtained for *Bam*HI experiments.

These data indicate that the strongest associations that can be detected at the human *IGF2-H19* locus in HB2 cells exist between the CTCF AD/DMR0 region and the CCD and CTCF DS sites, whereas a weak interaction may also exist between the ICR and CTCF AD/DMR0. The interaction between the CTCF AD/DMR0 and the ICR could be weaker than the CTCF AD/DMR0 and the CCD because the ICR is a monoallelic CTCF/cohesin binding site, and/or because our ChIP experiments indicated that less CTCF and cohesin are bound to the ICR than to the other sites (Figure 2A and 2B). Alternatively, we cannot exclude the possibility that the 3C interactions that we detected between the ICR and CTCF AD/DMR0 were caused indirectly by the strong associations between CTCF AD/DMR0 and the CCD and the CTCF DS sites, which are located to the left and the right of the ICR.

### Allele specificity of chromatin associations

**Figure 4:**
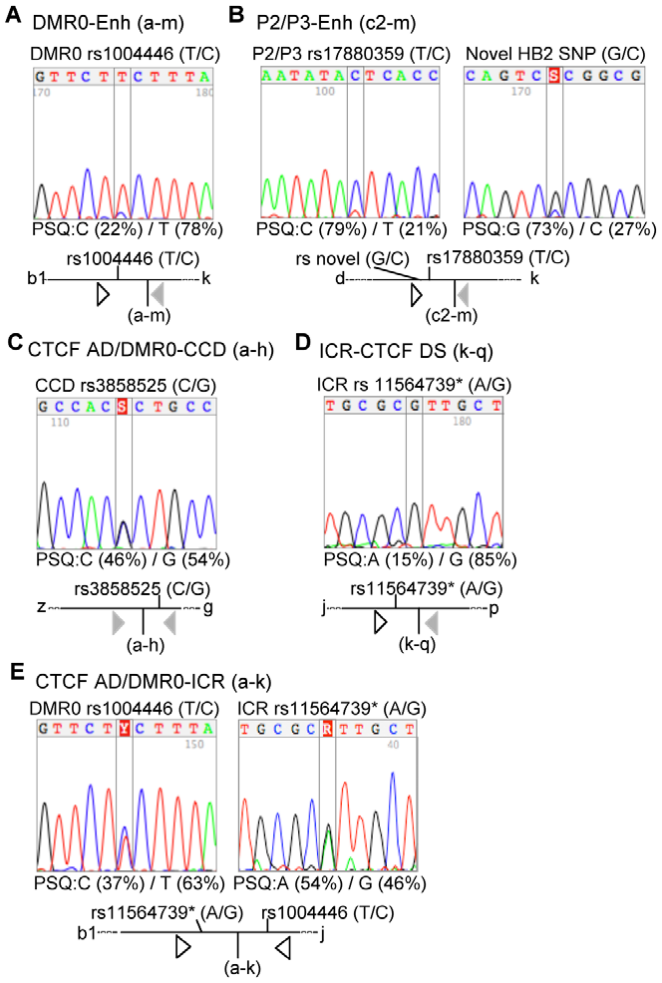
3C-SNP analysis. (A–E) show SNP sequencing results with electropherograms of the sequence and allele ratios by pyrosequencing (PSQ) analysis. A diagram indicating the 3C ligation product with the orientation of primers and SNPs is given below each sequence result. (A) 3C ligation product of restriction sites a and m which represents DMR0 and the enhancer showing preferentially monoallelic interaction at a SNP within fragment a-b1. The a-b1 fragment is close to the P0 and P2 promoters in the flanking *Bam*HI fragments. (Promoters can extend up to 5 Kb upstream of the transcription start site and the resolution of the 3C technique does not enable distinction between different promoters when they are in close proximity). (B) SNP analysis of 3C ligation product of restriction sites c2 and m representing an interaction between the enhancer and *IGF2* promoters near restriction site c2. Both SNPs in this region show monoallelic interactions. (C) Allelic analysis of 3C ligation product of restriction sites a and h, using a SNP near restriction site h in the CCD shows that associations between CTCF AD/DMR0 and CCD are biallelic. This SNP also showed that CTCF bound to both alleles at the CCD in Figure 2. (D) SNP analysis of 3C ligation product of restriction sites k and q indicative of an interaction between the ICR and the downstream CTCF DS showing a monoallelic interaction. (E) SNP analysis of 3C ligation products between ICR and the CTCF AD/DMR0 (restriction sites a–k). Both sides of the hybrid product show biallelic associations.

To examine which of the detected chromatin associations is allele specific we combined the 3C assays with SNP analysis of ligated products. Using polymorphisms in two separate restriction fragments, we detected associations between the enhancer and the *IGF2* promoter region predominantly on one allele (Figure 4A and 4B), which presumably represents the paternally derived allele. These promoter-enhancer interactions suggest that, despite the low levels of *IGF2* transcription, the chromatin conformation in HB2 cells is favourable for monoallelic expression. Associations between the CTCF AD/DMR0 region and the CCD could be detected on both alleles, consistent with our finding that CTCF and cohesin bind to CTCF AD sites and the CCD biallelically (Figure 4C). Monoallelic interactions were detected on the CTCF binding allele (presumably the maternal one) between the ICR and the CTCF DS (Figure 4D). Unexpectedly, we also detected biallelic associations between the ICR and the CTCF AD/DMR0 region (Figure 4E), despite the fact that CTCF binds the ICR predominantly on one allele. This result suggests that ICR- CTCFAD/DMR0 interactions are indirect and mediated through interactions between the CTCF AD/DMR0 with other CTCF sites near the ICR such as the CTCF DS. It is also conceivable that elements within the DMR0 and the adjacent CTCF sites interact separately with the methylated and the unmethylated ICR. However, most regulatory elements are quite close to the ICR and it is, therefore, also possible that the paternal promoter-enhancer interactions distort the conformation of the loop in a manner such that the paternal ICR may also be in close proximity to *IGF2*.

### Higher-order chromatin conformation is maintained in G1 and G2 phases of the cell cycle

Because our ChIP experiments revealed the strongest CTCF and cohesin signals at the CTCF AD, CCD and CTCF DS sites (Figure 2A and 2B), and our q3C assays had identified the strongest interactions between these sites, we hypothesised that these interactions could be mediated by cohesin. First, we tested two predictions that are made by this hypothesis. Cohesin associates with chromatin throughout interphase and regulates transcription at the *IGF2-H19* locus both in G1 and G2 phase [6]. If cohesin affects gene regulation by enabling the formation of chromatin loops one would, therefore, predict that these loops are also present in G1 and G2 phase. To test this we used 3C assays to compare chromatin conformation between cells that were synchronised by double thymidine arrest-release either in G1 or G2 phase. Figure 3 shows that synchronisation of cells in G1 and G2 phase did not change the overall 3C profiles using the CTCF AD/DMR0, ICR or enhancers as anchors. An exception was observed for the association between the ICR anchor and the CTCF DS which did not stand out as a “peak” in G1 cells, but could be detected as a “shoulder” in the 3C profile of the G2 cells. We presently do not know if this difference is of physiological relevance. Since most interactions did not change between G1 and G2 phase, our results are consistent with the possibility that cohesin mediates chromatin loop formation throughout interphase. Importantly, these data also suggest that cohesin's role in chromatin conformation would have to be independent of its function in sister chromatid cohesion, which exists in G2 but not G1 phase.

### Cohesin is associated with loops that are formed by interactions between CTCF sites

**Figure 5:**
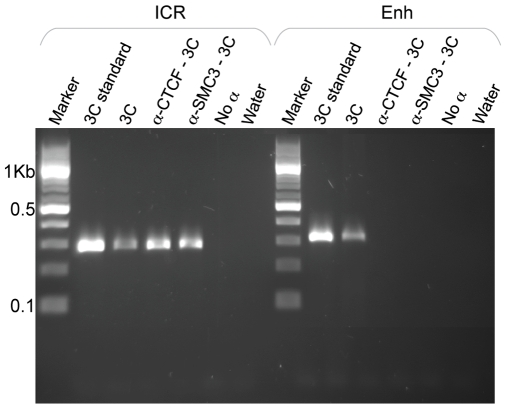
ChIP–loop experiments indicate that CTCF and cohesin are present at the interactions between CTCF AD*/*DMR0 and the ICR. Agarose gel showing a PCR product of 272 bp amplified with primers for the ICR and CTCFAD/DMR0 (primers at j and b1 restriction sites in Figure 1A) and a PCR product of 303 bp amplified with primers for the enhancer and CTCFAD/DMR0 (primers at m and b1 restriction sites in Figure 1A). Templates in each panel include 3C standard (template from PCR standard curve), 3C (cross-linked chromatin *Bam*HI digested and ligated), α-CTCF-3C, and α-SMC3-3C (ChIP–loop templates that were immuno-precipitated with CTCF or cohesin antibodies prior to ligation). ICR interactions are found in 3C and ChIP-loop material, likely reflecting a specific CTCF/cohesin mediated association between CTCF AD and the ICR, with both proteins present in the loop. In contrast, enhancer interactions are not found in ChIP–loop material confirming that this interaction is not CTCF/cohesin dependent and reflecting an association between the enhancer and the P2 promoter rather than with DMR0.

Another prediction made by the hypothesis that cohesin mediates 3C interactions between CTCF sites at the *IGF2-H19* locus is that cohesin should be present in the corresponding chromatin loops. Since our cohesin ChIP experiments (Figure 2A and 2B) did not distinguish between binding of cohesin to DNA molecules which were folded into loops and those that were not, we enriched the 3C digested templates for CTCF or cohesin bound chromatin before ligating for 3C analysis (ChIP-loop).We found that both SMC3 and CTCF antibodies can immunoprecipitate 3C products representing associations between the ICR (j restriction site) and the CTCF AD/DMR0 region (b1 restriction site) (Figure 5). These results indicate that cohesin is present in the corresponding chromatin loops. Importantly, 3C products representing enhancer-promoter associations could not be detected. These observations indicate that enhancer-promoter interactions cannot directly be mediated by cohesin, whereas the data are consistent with the possibility that cohesin mediates chromatin association between CTCF sites.

### Depletion of cohesin leads to destabilisation of chromatin conformation

**Figure 6:**
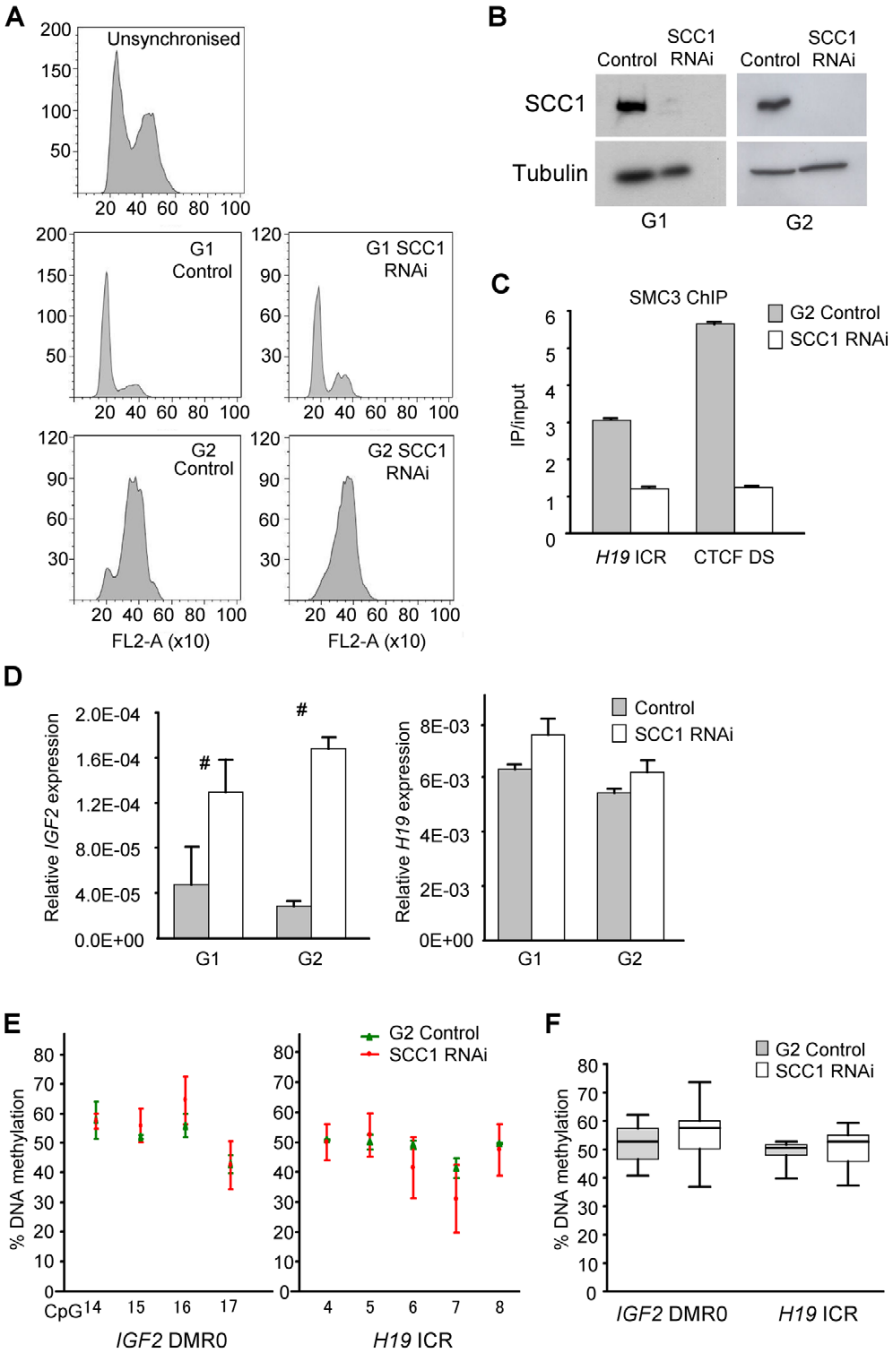
Cell-cycle synchronisation and siRNA depletion of cohesin. For cohesin depletion experiments, cells were transfected with RNAi against SCC1. RNAi against luciferase was used as a control. (A) Plots of FACS sorted cells synchronised in G1- and G2-phase in control and cohesin depleted cells. (B) Western blots showing SCC1 protein levels after RNAi treatment (C). SMC3 ChIP on G2 cells after cohesin depletion showing that cohesin is reduced at the ICR and the CTCF DS sites. (D) Expression levels of *IGF2* and *H19* relative to β-actin, as determined by real time PCR after cohesin depletion in G1 and G2 phases. *IGF2* is significantly upregulated while *H19* levels are unaffected. # Denotes significant differences (P<0.05) between control and SCC1 RNAi. (E) DNA methylation levels of individual CpGs in the DMR0 and ICR were determined by pyrosequencing of bisulphite modified DNA from cohesin depleted cells and controls in G2 phase. (F) Average DNA methylation levels at all CpGs detected by pyrosequencing in Figure 6E shows that methylation was not significantly changed after RNAi treatment for SCC1. Box and whisker plots show mean, inter-quartile ranges, max, and min values. Data represent triplicate bisulphite conversion and pyrosequencing reactions from one RNAi and control experiment.

To test directly if cohesin is functionally required for the formation or maintenance of chromatin loops at the *IGF2-H19* locus, we depleted the cohesin subunit SCC1 by RNAi and thus rendered the cohesin complex non-functional. Since cohesin depleted cells delay progression through mitosis, and because it is unknown if chromatin loops are maintained during mitosis, we synchronised SCC1 depleted cells by double thymidine treatment and harvested cells in G1 and G2 phases (Figure 6A and 6B). We confirmed that CTCF was still bound to the ICR, and the CCD after cohesin depletion in HB2 cells (results not shown), as was previously demonstrated in another cell line [6]. The effect of depletion of SCC1 on *IGF2* and *H19* expression was an activation of *IGF2* transcription, but no significant difference in *H19* expression (Figure 6D). Average DNA methylation levels at the *IGF2* and *H19* DMRs did not change significantly (Figure 6E and 6F).

**Figure 7:**
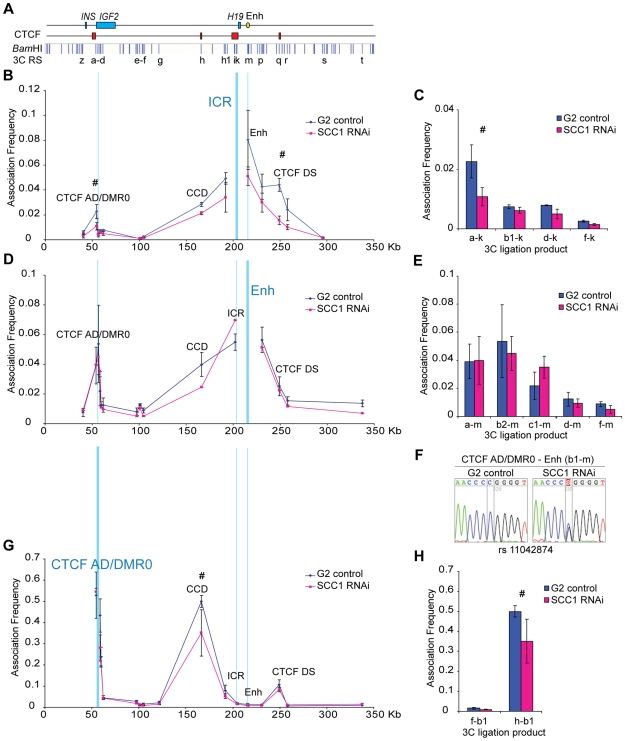
Depletion of cohesin by siRNA results in ablation of the CTCF-mediated loops. (A) Schematic representation of a 350 Kb genomic region including *INS* and the *IGF2-H19* locus as shown in Figure 3. (B) Associations detected with an anchor site in the ICR (primer k) in G2 phase cells treated with control RNAi (G2 control) or RNAi against the cohesin subunit SCC1 (SCC1 RNAi). Association between the ICR and CTCF/cohesin sites, i.e. CTCF AD, CCD, and CTCF DS, are reduced in SCC1 depleted cells indicating a general “loosening” of the chromatin structure. (C) Histogram of association frequencies between ICR anchor and restriction sites at the CTCF AD/DMR0 peak. (D) Enhancer associations (m primer) after cohesin RNAi are not generally reduced. Reduction at the intervening CCD and ICR sites may be due to the influence of disrupted associations at CTCF/cohesin bound sites. (E) Histogram of association frequencies between Enh anchor and restriction sites at the CTCF AD/DMR0 peak. (F) Sequencing of b1-m interaction shows a change from mono-allelic interactions in controls to biallelic interactions after cohesin depletion. (G) Association frequencies of CTCF AD/DMR0 (restriction site b1 as anchor) show a significant reduction in the association with CCD. (H) Histogram representing reduction in CTCF AD/DMR0 -CCD (h-b1) association after cohesin depletion. Table S1 has statistical analysis of all interactions between anchor primers and *Bam*HI sites. # Denotes significant differences (P<0.05) between control and SCC1 RNAi.

In SCC1 depleted cells that were enriched in G2 phase we found a significant reduction in the association frequency between all chromatin interactions between CTCF binding sites, with the exception of the interaction between the CTCF AD/DMR0 region and the very distant CTCF DS sites (Figure 7B, 7C, 7G, and 7H and Table S1). Similar results were obtained using an ICR anchor primer in another restriction site within the ICR (Primer j, Figure S3). Using *Bgl*II as a restriction enzyme we were also able to confirm that there is a 30% reduction between the CTCF AD and CCD interactions during G2 phase (Figure S2D). Importantly, however, associations with the enhancer anchor (primer m) and the *IGF2* promoters (restriction sites b2, c1) were not significantly reduced (Figure 7D and 7E). These observations indicate that 3C interactions between CTCF sites are dependent on cohesin, whereas the ability of the enhancer to associate with the *IGF2* promoters is not dependent on cohesin. Importantly, when we sequenced the PCR products from the enhancer anchor, we found that the monoallelic associations found in the controls became biallelic after cohesin depletion (Figure 7F).

SCC1 depleted cells harvested in G1 phase yielded less chromatin for analysis than that in G2 phase and locus wide comparisons could not be done. However we were able to show that in G1, associations between the ICR (j primer) and the CTCF AD/DMR0 regions (restriction sites a- c1) were also reduced (Figure S4). Together, these results indicate that depletion of cohesin predominantly affects CTCF mediated looping interactions. Importantly, our observation that cohesin depletion also affects chromatin structure in G1 phase, where no sister chromatid cohesion exists, further supports the notion that cohesin's role in chromatin looping is independent of its cohesion function.

**Figure 8:**
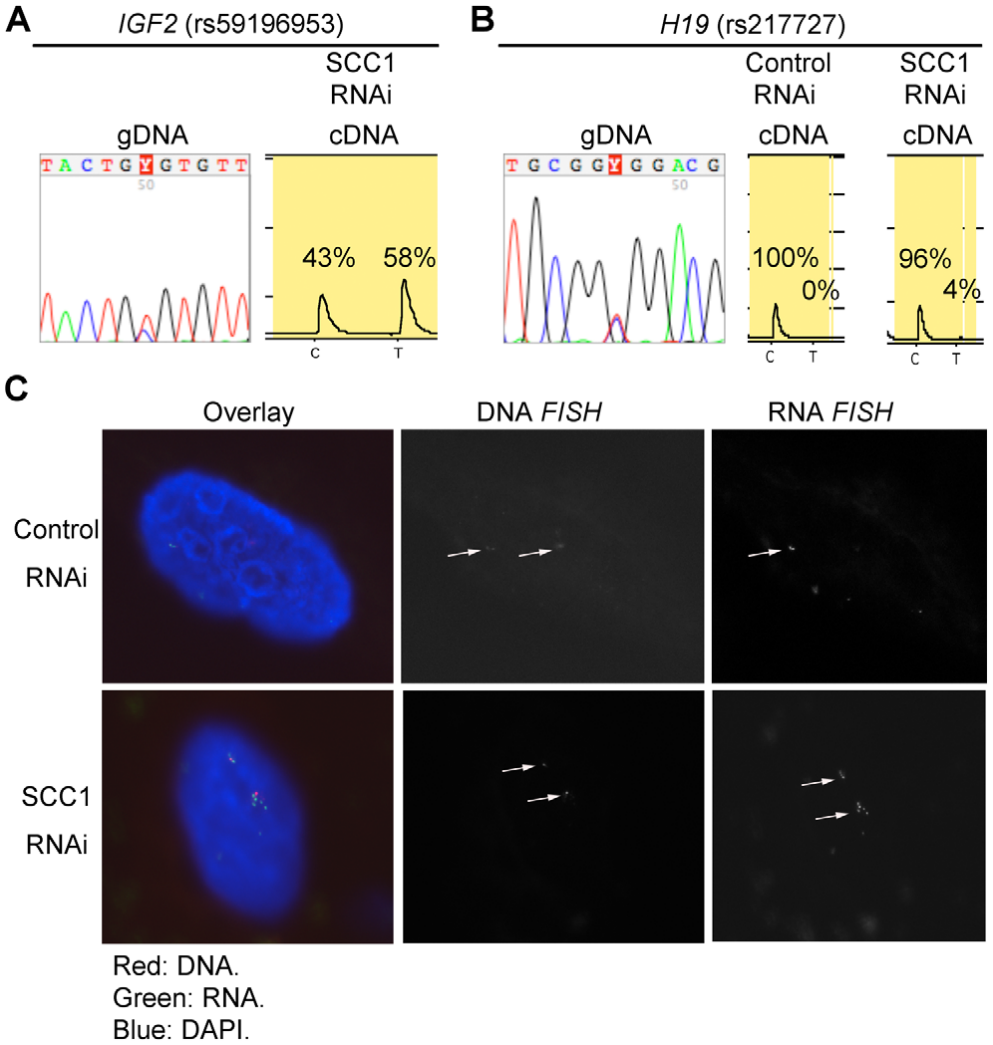
Biallelic *IGF2* expression after cohesin depletion. (A) Sequencing of genomic DNA (gDNA) shows that the HB2 cell line is heterozygous for a SNP in *IGF2*. Pyrosequencing of cDNA of cohesin depleted G2 phase cells shows both *IGF2* alleles indicating biallelic *IGF2* expression. (B) Sequencing of genomic DNA (gDNA) shows that the HB2 cell line is heterozygous for a SNP in *H19*. Pyrosequencing of cDNA of cohesin depleted and control G2 cells shows only the C allele indicating that *H19* expression remains monoallelic after cohesin depletion. (C) After labelling *IGF2* RNA by RNA fluorescence in situ hybridisation (FISH; green) on cohesin depleted (SCC1 RNAi) and control interphase HB2 cells (RNAi control), DNA FISH (red) was performed to detect genomic *IGF2* sequence. Nuclei were counterstained with DAPI (blue) and imaged by confocal microscopy. Representative images are shown; first panel: overlay, second panel: DNA signal, third panel: RNA signal. *IGF2* expression increases and becomes biallelic upon cohesin depletion.

The effect of decreased CTCF and cohesin mediated 3C associations had little or no impact on imprinted expression of *H19* which remained mono-allelic (Figure 8B), presumably due to the methylation being maintained at the ICR (Figure 6E and 6F). Expression of *IGF2* was biallelic after cohesin depletion as determined by SNP analysis and RNA FISH (Figure 8A and 8C). However, *IGF2* expression levels were so low prior to cohesin depletion that was impossible to tell whether the basal transcription was monoallelic or biallelic.

## Discussion

It is now well established that cohesin complexes do not only function in sister chromatid cohesion but also have important roles in gene regulation, both in proliferating and post-mitotic cells (reviewed in [3],[37]). However, it remains largely unknown how cohesin mediates these effects. Our results suggest that cohesin contributes to gene regulation by mediating the formation of higher order chromatin conformation, at least at the imprinted *IGF2-H19* locus. Recent studies of the apolipoprotein cluster [38] and of the interferon γ locus [39] have revealed that cohesin also has roles in long-range chromatin interactions at these loci. It is, therefore, possible that cohesin has a widespread role in the formation of chromatin loops in mammalian genomes and regulates gene expression through this mechanism at numerous sites. In our experiments 3C interactions were reduced but not abolished after cohesin depletion. It is possible that the remaining chromatin interactions were caused by residual amounts of cohesin, which is difficult to deplete completely by RNAi. However, we can not exclude the possibility that other proteins maintain the chromatin loops in the absence of cohesin.

Our first systematic q3C analysis of the human *IGF2-H19* locus has brought to light an unexpected complexity of chromatin interactions. We found evidence for *IGF2* promoter-enhancer interactions on the putative paternal allele [17],[18] as well as ICR interactions with CTCF sites at the 5′ end of the *IGF2* gene (CTCF AD sites). Previous studies in mice have identified allele specific interactions of the ICR and the enhancers, but CTCF sites other than the ICR have not yet been analysed at the mouse locus. By extending our analysis to a wider number of CTCF sites we found a previously unknown association of the ICR with a CTCF DS site on the presumed maternal allele in the human cell line, as well as biallelic CTCF mediated interactions involving the CCD site. The CCD region in mice has tissue specific silencer or enhancer activities which are independent of imprinting [26],[27],[30]. Genome-wide CTCF ChIP-sequencing data in adult mouse livers indicate that this region also binds CTCF (D. Odom and D. Schmidt, pers. communication), suggesting that the CCD may function as a boundary or insulator with regard to its silencer function. It is thus possible that cohesin is also required for the insulator activity of the CCD.

**Figure 9:**
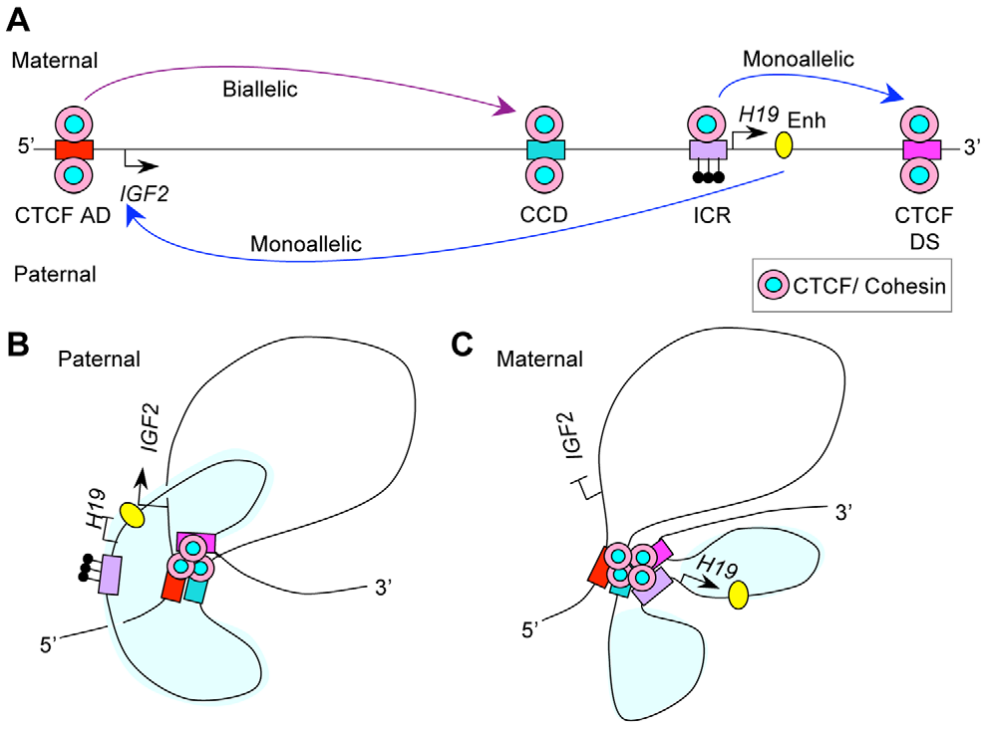
Simplified model of the cohesin and CTCF–mediated interactions in the human *IGF2-H19* locus. DNA elements are indicated as follows: CTCF AD (red bar); CCD (green bar), ICR (purple bar), and CTCF DS (Cerise bar), Enhancer is yellow oval. Pink and pale blue ovals represent the CTCF/cohesin complexes. CpG methylation is depicted as filled lollipops. (A) Linear representation of the *IGF2-H19* locus. Elements above the bar represent the maternal allele with CTCF and cohesin binding the ICR and an active *H19* gene. Elements below the bar represent the paternal allele with active *IGF2* gene and methylated ICR. ChIP data indicate that cohesin and CTCF co-localise at the CCD, CTCF AD and CTCF DS on both alleles. 3C data indicate that these CTCF/cohesin sites interact strongly with each other; while the ICR and enhancer have limited allele specific interactions (long curved arrows indicate 3C interactions between 3C elements). Based on these data we propose the following model: (B) On the paternal allele, co-localisation of CTCF and cohesin at CTCF AD, CCD, and CTCF DS brings these regions together. The methylated ICR does not bind CTCF and is thus excluded from CTCF/cohesin interacting regions. The exclusion of the ICR may enable the *IGF2* gene promoters and *H19* enhancer region to interact, (shown by yellow oval close to *IGF2* arrow) even though they are on different looping domains. (The *H19* domain is shaded.) (C) On the maternal allele, CTCF/cohesin can bind to the unmethylated ICR which can then interact with other CTCF/cohesin sites. An interaction between CTCF AD/DMR0 and the ICR may be indirectly mediated through the interaction between CTCF AD/DMR0 and CTCF DS. A monoallelic interaction between the ICR and CTCF DS could redefine the H19 domain and constrain the enhancer to prevent interaction with *IGF2* promoters on the maternal allele. Without cohesin, CTCF does not maintain stable loops and *IGF2* promoters can access the enhancers and perhaps even other regulatory elements from neighbouring genes. Interactions between the various CTCF sites are likely to be dynamic and may occur sequentially.

Our data are consistent with the possibility that multiple CTCF-cohesin mediated loops come together in a chromatin “hub” as depicted in Figure 9. At this hub CTCF and cohesin might bring various regulatory elements into close proximity to enable interactions between distant elements, either simultaneously as is drawn in our model, or possibly in a sequential order. Cohesin may stabilise the CTCF mediated interactions. Cross-linked chromatin enables us to study a snapshot of interactions at any given time, but it is likely that these interactions are dynamic with some occurring more rapidly than others.

Transcription of *IGF2* and *H19* is developmentally down regulated in most adult tissues but reactivated in various cancers (reviewed in [13]). We chose a normal epithelial breast cell line to study the conformation of the adult *IGF2-H19* locus. After the disruption of CTCF mediated chromatin conformation by cohesin depletion *IGF2* expression was reactivated in these cells. Moreover, substantial biallelic expression of *IGF2* was observed and enhancer-promoter associations changed from mono- to biallelic. Interestingly, biallelic *IGF2* expression was not accompanied by hypermethylation at the ICR, suggesting that depletion of cohesin can uncouple the relationship between *IGF2* expression and methylation at the *H19* ICR. Methylation profiles of the *IGF2-H19* locus in many cancers indicate that loss of *IGF2* imprinting and methylation are often disconnected during neoplasia [40]–[46]. The roles of higher order chromatin structure and loss of imprinting in cancer are still largely unexplored. Defects in proper positioning of cohesin on DNA could therefore contribute to abnormal gene regulation in neoplastic cells.

The finding that cohesin is required to stabilise higher order chromatin conformation raises the intriguing possibility that cohesin physically connects two DNA sequences on the same DNA molecule in *cis*, to form loop structures similar to how cohesin interacts with two DNA molecules in *trans*, to mediate sister chromatid cohesion. It is conceivable that cohesin forms chromatin loops by embracing two DNA strands at the base of a loop, similar to how cohesin has been proposed to mediate cohesion as a ring [2]. Alternatively, it is possible that cohesin complexes bound to two DNA sites can interact with each other, as has been suggested for CTCF molecules [47]. Our finding that cohesin depletion interferes with chromatin loop formation, although cohesin depletion does not abrogate CTCF binding [6], supports our hypothesis that one of CTCF's main roles as a transcriptional insulator may be to recruit cohesin to insulator sequences. Hypomorphic mutations in the cohesin subunits SMC1 and SMC3 and in the cohesin loading factor NIPBL have been identified as the molecular cause of Cornelia de Lange syndrome, a rare human developmental disorder [48]–[50]. The results in this study show a modest reduction in looping interactions after cohesin depletion which suggest that cohesin is a stabilising factor in chromatin looping. It will therefore be important to test if these mutations affect higher order chromatin structure at specific loci. Although such defects may be very subtle, they could at some loci cause defects in gene regulation during development.

Another important goal for the future will be to determine at the genomic level which CTCF-cohesin sites can interact with each other, if these interactions change during cell differentiation and how such changes might be specified.

## Methods

### *IGF2-H19* methylation and expression analysis

Methylation analysis was by bisulphite- and pyrosequencing with primers as described previously [23],[25],[51]. Expression analysis was done by qPCR on reverse transcribed RNA. Primers for qPCR were *IGF2* Fwd CTCACCTTCTTGGCCTTCG, *IGF2* Rev GGAAACAGCACTCCTCAACG, *H19* t Fwd GAGATTCAAAGCCTCCACGACT and *H19* Rev GCGTAATGGAATGCTTGAAGG. B Actin was analysed using primers from a QuantiTect Assay (Qiagen). Quantitation was done by extrapolation to standard curves for the pimers. Wilcoxon signed rank tests were done to compare paired RNAi and control samples when n≥3. P≤0.05 was considered significant.

### ChIP

Chromatin Immunoprecipitation (ChIP) was done as described previously [6]. Input and immunoprecipitated (IP) material was quantified by Picogreen (Invitrogen), and real-time PCR with standard curves was performed. Values were corrected for DNA amount, and enrichment was calculated as IP over input. When comparing ChIP from cohesin depleted cells with control cells (Figure 6C), the IP/Input was further normalised against a region where CTCF does not bind (*IGF2* exon 9). ChIP primers are shown in Table S2.

### 3C

Quantitative 3C was described previously [31],[36],[52],[53] and performed with the following modifications: 5×10^6^ of cells were cross-linked in 1% formaldehyde at 37°C for 10 minutes. After washing in PBS, the cells were lysed on ice in lysis buffer (Tris-HCl (pH = 8) 50 mM, SDS 1%, EDTA 10 mM) for 10 minutes. Nuclei were recovered by centrifugation and resuspended in *Bam*HI digestion buffer (New England Biolabs (NEB)), supplemented with Triton-X100 to a final concentration of 1.8% and incubated 1 h at 37°C. 1.5×10^6^ nuclei were digested overnight with 1000 U of *Bam*HI (NEB) in a 300 µl reaction volume.

Digestion efficiency at each *Bam*HI restriction site within the locus was assessed by qPCR across each restriction site. The percentage of digestion was determined by comparing template amplification of digested and undigested fractions (not religated) after normalising to copy number as previously described [31]. All regions within the locus were digested equally efficiently. This step was an important quality control check, and if digestion was below 70% the chromatin was discarded.

Ligation was carried out on 2.5 ng/µl digested chromatin in a 1.5 ml reaction volume of T4 ligase buffer containing 3200 U of T4 ligase (NEB). A further overnight digestion step with 1000 U of *Eco*RI (which cuts outside the hybrid religated products) was incorporated prior to reversal of cross links, phenol chloroform purification and ethanol precipitation. This step is necessary to remove possible qPCR bias caused by size differences in the religated products.

3C PCR primers flanking restriction sites were designed to have similar melting temperatures, and the PCR efficiency of each primer combination was assessed on a PCR standard template. A stock of PCR standard template was prepared similar to that described previously [52] by amplification of 36 genomic regions across the *IGF2-H19* locus on commercially obtained genomic DNA (Becton Dickinson (BD)). These amplicons were column purified and quantified using Nanodrop UV spectroscopy. Equimolar amounts of amplicons were mixed, *Bam*HI digested, re-ligated, phenol-chloroform extracted, ethanol precipitated, dissolved in H2O and stored at −20°C. Q-PCR was done with Sybr-green (ABI Power SYBR) on a 384 well real time machine (7900HT Fast Real time PCR system, Applied Biosystems). Quantitative determination of association frequencies was essentially done as described [31]. Copy number of 3C template was determined by qPCR amplification of a region between *IGF2* and *H19* which did not have *Bam*HI restriction sites (Chr11: 2057922–2057991, Ensembl). Template copy number was used to ensure that the amount of 3C template was within the range of the standard curve for any given product.

All interaction frequencies were normalised to the circularisation frequency of the i-fragment as internal digestion-ligation control. We verified our normalisation method by including a β-actin gene region (Chr7: 5,326,283–5,357,206) that contained 3 *Bam*HI restriction sites and compared the outcome of the 3C association frequencies across the locus when normalised to adjacent *Bam*HI sites (2–3 B-actin), alternative *Bam*HI sites (1–3 B-actin), or alternative internal sites within the *IGF2-H19* locus (data not shown). 3C primers and combinations are in Table S2 and S4.

Biological replicates for siRNA 3C experiments were done by splitting test and control cells each into 3 equal aliquots prior to synchronisation. After harvesting the cells and digesting the chromatin for 3C experiments, the replicate templates were evaluated for digestion efficiency and normalised for equal amounts before ligation. Prior to PCR amplification, the replicate templates' copy numbers were determined by qPCR as described above, and equal amounts (copy number) of DNA recovered after 3C for control and RNAi template was used for the 3C qPCR. Association frequencies were normalised to the circularisation frequency of the i-fragment as described above. Normalisation data for 3 biological replicates are shown in Figure S5. The frequency of circularisation of the i-fragment is similar in controls and RNAi treated cells (Figure S5A), confirming that RNAi treatment for cohesin depletion did not affect digestion or ligation efficiency. Using the circularisation frequency of the i-fragment to normalise an association frequency between the enhancer anchor (primer m) and a restriction site located between the enhancer and CTCF DS (restriction site p), shows no significant differences between RNAi treated cells and controls (Figure S5B). This is as expected, because there is no binding of CTCF or cohesin to the enhancer and its interaction with restriction site p is due to random ligation. Circularisation of the i-fragment was therefore a suitable internal normaliser.

### ChIP loop

ChIP loop was performed as follows: 3 aliquotes of 5×10^6^ cells were first fixed in formaldehyde and nuclei were prepared as for 3C. One aliquot was used for 3C and the remaining aliquots were briefly sonicated to produce chromatin fragments of 500 bp and then digested overnight with *BamH1* as for 3C protocol. After digestion the nuclei were pre-cleared on agarose beads (UPSTATE) and immunoprecipitated with antibodies to cohesin (anti-SMC3 [6],[54] and CTCF (UPSTATE) as described in the ChIP protocol. After washing in ChIP washing buffer (UPSTATE), the beads and antibody complexes were resuspended in 1.5 ml of 3C ligation mix, containing 3200 U of T4 ligase, overnight at 15°C. After ligation, the samples were purified as in the 3C protocol. After 40 cycles of PCR, bands were visualised on an agarose gel and compared to the band obtained with 3C.

### RNAi depletion and cell-cycle synchronization

RNAi knockdown of cohesin was done as described previously [6]. To obtain cells enriched in G2 and G1 phase HB2 cells were synchronised by double thymidine block: addition of 3 mM thymidine for 16 h, removal of thymidine by washing with PBS and release of the cells from the block for 8 h, addition of 3 mM thymidine for another 16 h for the second block. The cells are released from the second block by washing with PBS and cells are harvested after 6 h for enrichment in G2 phase and after 14 hours for enrichment in G1 phase. The enrichment of the cells in the respective cell cycle phases was controlled by FACS.

To obtain cells enriched in G1 and G2 phase and depleted of the cohesin subunit SCC1 the siRNA transfection was performed either 6 hours before starting the first thymidine block (for G2 phase) or 2 hours after releasing the cells from the first thymidine block (for G1 phase). The siRNA oligos (sense-GGUGAAAAUGGCAUUACGGtt and antisense CCGUAAUGCCAUUUUCACCtt, Ambion) were annealed according to manufacturer's instruction and used at a final concentration of 75 nM. The siRNA transfection was performed using lipofectamine RNAiMAX (Invitrogen).

### Statistical analysis of 3C after RNAi

Two-way ANOVA with Bonferroni's post-test was performed using GraphPad Prism version 5.01 for Windows, GraphPad Software, San Diego California USA, www.graphpad.com. Normalised values of 3 biological replicate experiments for each ligation combination in a given anchor set was analysed by two-way ANOVA, with RNAi/control being one set of factors and restriction sites being the other set of factors. A Bonferrroni post post-test enabled comparison of multiple replicates at each restriction site. The Bonferroni correction lowers the P value considered significant to 0.05 divided by the number of comparisons. Thus in *n* rows of data with two columns (Control and RNAi), the P value has to be less than 0.05/n, for any particular row in order to be considered significant with P<0.05. This correction ensures that the 5% probability applies to the entire set of comparisons, and not separately to each individual comparison.

### RNA and DNA FISH

To obtain a probe for RNA FISH two PCR products of 2000 bp and 600 bp (primer sequences available upon request) were generated from the last exon of the human *IGF2* gene, mixed and labeled with dig-11-dUTP using the Biotin High Prime Kit (Roche). For the DNA probe the human BAC RP11-650021 spanning the *IGF2* gene (chr.11: pos. 2057305–2245714) was directly labeled with Alexa 594 by random priming. Cells on coverslips were fixed for 15 min with 4% formaldehyde, 5% acetic acid in PBS and stored after another PBS wash in 70% ethanol at 4°C.

Denaturation and hybridisation of the slides and probes was done as described in [54]. The biotin labelled probe was detected by successive incubations with mouse-anti biotin (DAKO, 1∶500 dilution) and FITC-conjugated goat-anti-mouse antibodies (Jackson ImmunoResearch Laboratories Inc., 1∶500 dilution) and after dehydration mounted using Vectashield with DAPI (Vector Laboratories). The slides were analysed on a Leica DMRBE microscope equipped with a Hamatsu CCD (C4880) camera with a 100X objective. Adobe Photoshop was used to colour the images and generate the overlay figures.

## Supporting Information

Figure S1 Schematic representation of *Bgl*II restriction sites at the *IGF2-H19* locus. (A) *INS*, *IGF2* and the *H19* gene are displayed together with the cohesin/CTCF binding regions. Blue boxes represent genes and the green oval represents the downstream enhancer (Enh). Red rectangles indicate the positions of the CTCF/cohesin binding regions CTCF AD, CCD, ICR, and CTCF DS. Vertical blue lines in a row show *Bgl*II restriction sites across the whole locus. Sites that were analysed for ligation (3C RS) are labelled with numbers. The third line shows the position of specific regions analysed for 3C and these are enlarged in the panels below. Note that CTCF AD has a restriction site 3 Kb far from DMR0 and can be analysed separately from other sites in the *IGF2* locus. In panels B-E *Bgl*II restriction sites are depicted as red vertical lines relative to *Bam*HI restriction sites shown as black lines and labelled with letters as in Figure 1. Black arrows and grey arrowheads indicate positions of anchor- and 3C- primers respectively. (B) Enlarged region encompassing the CTCF AD, the DMR0 and the *IGF2* gene showing exons as boxes (blue for coding- and white for non-coding exons). Promoters are shown as arrows above the exons. The DMR0 region is shown as a yellow bar. (C) Enlargement of the intervening region between *IGF2* and *H19* showing the position of the CCD (red box) relative to the *Bgl*II and *Bam*HI restriction sites. (D) Enlargement of ICR and *H19* region. The CTCF/cohesin binding region is shown as red box and the position of the ICR is marked with a yellow box. (E) Enlargement of the enhancer and the CTCF DS region (green oval and red box respectively).
(0.66 MB TIF)

Figure S2 Chromatin conformation of the *IGF2-H19* locus using *Bgl*II restriction. (A) Schematic representation of a 350 Kb genomic region including *INS* and the *IGF2-H19* locus. Blue boxes represent the position of genes and the green oval depicts the localisation of the enhancer (Enh). Red boxes below represent the CTCF/cohesin binding regions CTCF AD, CCD, ICR, and CTCF DS. Positions of *Bgl*II restriction sites are marked with vertical blue lines and sites analysed by 3C are showed with numbers. Vertical pink lines in panel (B–D) indicate the position of the two different *Bgl*II restriction sites that were used as anchors. In each panel the respective anchor is marked with a thicker pink line. The X-axis is labelled according to the genomic position and position 0 was arbitrarily fixed 42 Kb upstream of *INS*. In (B,C) unsynchronised cells were analysed for the 3C interactions of the locus. (B) Associations detected with the anchor site in the ICR (restriction site 22 as anchor). The ICR associates with the CTCF AD and also with P2. (C) Associations with the CTCF AD (restriction site 4 as anchor). Interactions can be detected with the CCD and CTCF DS. (D) CTCF AD association frequencies (restriction site 4 as anchor) in cohesin depleted (SCC1 RNAi) and control (G2 control) cells synchronised in G2 phase. Interactions can be detected with the CCD and CTCF DS. Associations with the CCD were significantly decreased after SCC1 RNAi while interactions with the CTCF DS are only mildly affected. These results are similar to that obtained in Figure 7 with *Bam*HI enzyme.
(0.64 MB TIF)

Figure S3 Chromatin conformation of the *IGF2-H19* locus using an alternative anchor site in the ICR. (A) Schematic representation of a 350 Kb genomic region including *INS* and the *IGF2/H19* locus. Blue boxes represent the position of genes, the green oval depicts the localisation of the enhancer (Enh) (see also Figure 1) and red boxes in the second line represent the CTCF/cohesin binding regions CTCF AD, CCD, ICR, and CTCF DS. Vertical blue lines in a row indicate the position of *Bam*HI restriction sites within the locus and letters point out the restriction sites analysed by 3C (3C RS) in the graphs and histograms shown in the other panels. The blue vertical line across each graph indicates the position of the anchor restriction site (j) in the ICR. (B,C) Association frequencies of the ICR (anchor site j) throughout the locus are analysed in unsynchronised cells as well as in cells synchronised in G1 and G2 phases. Association frequencies are displayed on a log scale to include all data points (B) but also on a linear scale (C) to better visualise differences. For all three cell populations, associations of the ICR can be detected with the CTCF AD/DMR0 region as well as with the CTCF DS. The CCD is too close to the ICR to distinguish an interaction. ICR association frequencies with intervening regions between the CCD and the *IGF2* gene (restriction sites e, f and g) vary between G1 and G2 phase. Since these are random ligation interactions we don't know what this means. We do not see this with an anchor near the k restriction site (Figure 3). The association frequencies between ICR and the CTCF AD/DMR0 were similar; however we do see a higher association frequency in G2 compared to G1 at the CTCF DS region. We also see this difference when we use the k primer as an anchor (Figure 3). The high interaction with the d restriction site is not reproducible. (D) Associations of the ICR with individual restriction sites close to the CTCF AD/DMR0 are depicted as histograms for higher resolution. (E,F) Association frequencies of the ICR (anchor site j) throughout the locus are analysed in cohesin depleted cells (SCC1 RNAi) or cells treated with control RNAi. Association frequencies are first displayed on a log scale to include all data points (E) but also on a linear scale in (F) to better visualise differences. All associations detected within the locus are reduced after SCC1 depletion. Statistical evaluation for all interactions with the j primers are in Table S1. (G) For a detailed view of ICR interactions with the CTCF AD/DMR0 region, effects of cohesin depletion are displayed as a histogram. # Denotes significant differences (P<0.05) between control and SCC1 RNAi.
(1.13 MB TIF)

Figure S4 Effect of cohesin depletion on chromatin conformation in the *IGF2/H19* locus in G1 phase cells. (A) Schematic representation of a 350 Kb genomic region including *INS* and the *IGF2-H19* locus. Blue boxes represent the position of genes, the green oval depicts the localisation of the enhancer (Enh) (see also Figure 1) and red boxes in the second line represent the CTCF/cohesin binding regions CTCF AD, CCD, ICR, and CTCF DS. Vertical blue lines in a row indicate the position of *Bam*HI restriction sites within the locus and letters point out the restriction sites analysed by 3C (3C RS). Anchor primers are shown as black arrows below the restriction site map. (B) Enlarged view of the region comprising the CTCF AD/DMR0 and the *IGF2* gene showing exons as boxes (blue for coding and empty for non-coding exons). Promoters are shown as arrows above the exons and the DMR0 region is shown as a yellow bar. Reciprocal 3C primers are shown as grey arrowheads. (C) Association frequencies of the ICR (j-primer as anchor) with restriction sites in the CTCF AD/DMR0 (a and b1) and *IGF2* gene promoter (c1) in cohesin depleted (SCC1 RNAi) and control (G1 control) cells synchronised in G1 phase. Association frequencies with restriction sites in *IGF2* upstream region drop significantly in cohesin depleted cells compared to a random ligation with a restriction site in the intervening region (restriction site e) that is not reduced. (D) The effect of cohesin depletion on associations between the enhancer (m primer) and restriction sites close to promoters P2 (b1 restriction site), and P3 (d restriction site), was analysed. Upon cohesin depletion no significant change in the association frequencies was observed, confirming that these interactions occur independently of CTCF/cohesin binding (Table S1). # Denotes significant differences (P<0.05) between control and SCC1 RNAi.
(0.74 MB TIF)

Figure S5 Effects of normalisation using circularisation of a restriction fragment as an internal control. *Bam*HI restriction of i and j sites followed by random ligation results in circularisation of this fragment (i-fragment). Circularisation of i-fragment can be detected by using primers amplifying 180 bp spanning the re-ligated i and j sites. (Primer sequences are located in Table S3.) Inefficient digestion or ligation will give very low yields of this PCR product and therefore it is a good ligation and digestion control. (A) We found that the variation of i-fragment circularisation relative to the copy number of template between 3 biological replicates was low. There was also no significant difference in the relative circularisation frequency of i-fragment between control and cohesin depleted samples. (B) All interactions in our 3C samples were normalised using the circularisation frequency of the i-fragment. There was no significant difference between control and cohesin depleted samples at an interaction between the enhancer anchor (primer m) and a restriction site between the enhancer and the CTCF DS (site p). The interaction between m and p is not dependent on CTCF/cohesin and is therefore not expected to change after cohesin depletion.
(0.29 MB TIF)

Table S1 Statistical analysis of difference in locus-wide association frequencies with ICR, enhancer, CTCF AD/DMR0, and CTCF DS anchors.
(0.31 MB DOC)

Table S2 ChIP primers.
(0.04 MB DOC)

Table S3 3C primers for *Bam*H1 template.
(0.12 MB DOC)

Table S4 3C primers for *Bgl*II template.
(0.06 MB DOC)
