# Supplementary figures and images for: Cohesin Is Required for Higher-Order Chromatin Conformation at the Imprinted IGF2-H19 Locus
Source: PLoS Genet. 2009 Nov 26;5(11):e1000739. doi: 10.1371/journal.pgen.1000739 (PMC2776306; doi:10.1371/journal.pgen.1000739)

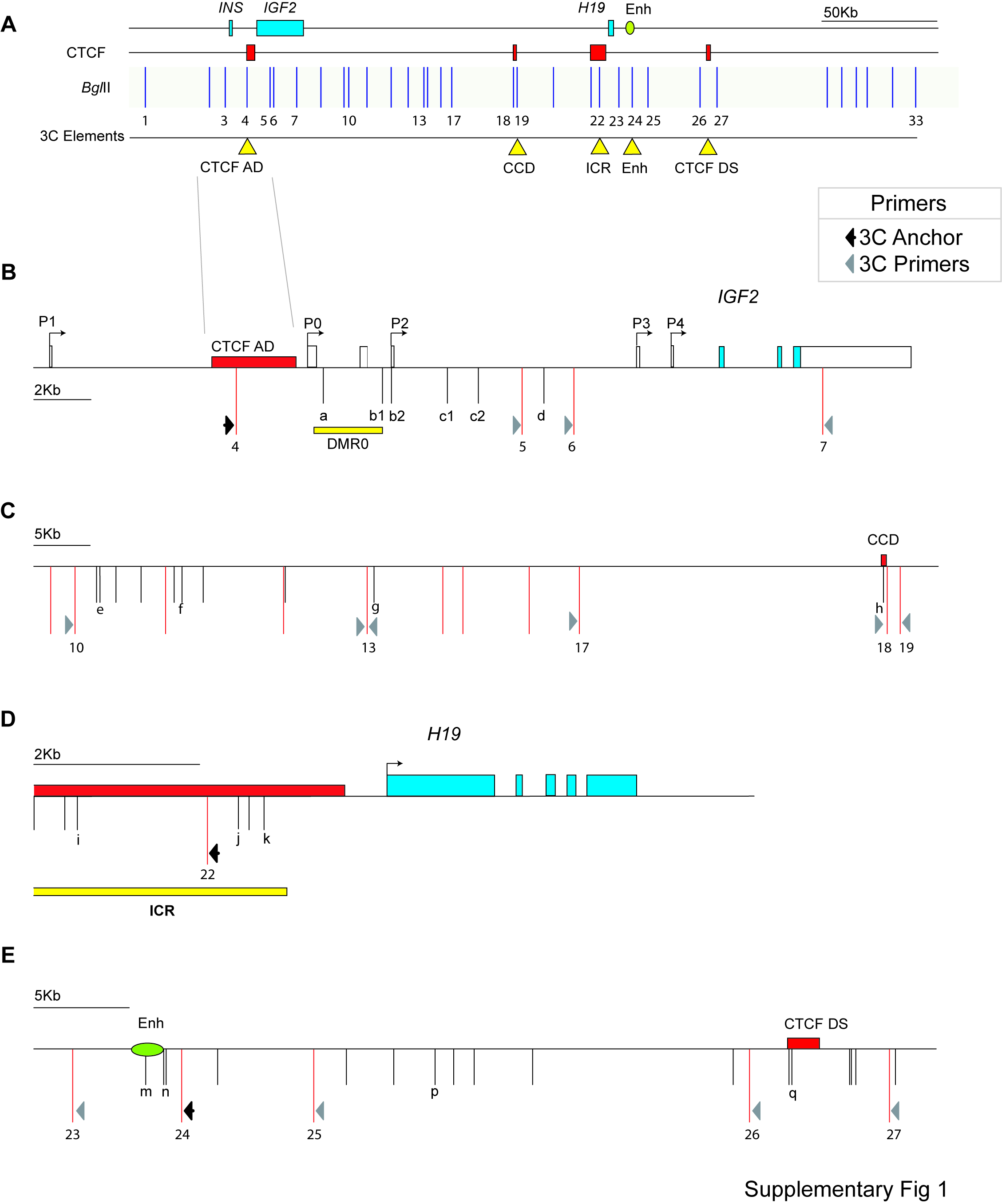

Supplement: Figure S1 — Schematic representation of BglII restriction sites at the IGF2-H19 locus. (A) INS, IGF2 and the H19 gene are displayed together with the cohesin/CTCF binding regions. Blue boxes represent genes and the green oval represents the downstream enhancer (Enh). Red rectangles indicate the positions of the CTCF/cohesin binding regions CTCF AD, CCD, ICR, and CTCF DS. Vertical blue lines in a row show BglII restriction sites across the whole locus. Sites that were analysed for ligation (3C RS) are labelled with numbers. The third line shows the position of specific regions analysed for 3C and these are enlarged in the panels below. Note that CTCF AD has a restriction site 3 Kb far from DMR0 and can be analysed separately from other sites in the IGF2 locus. In panels B-E BglII restriction sites are depicted as red vertical lines relative to BamHI restriction sites shown as black lines and labelled with letters as in Figure 1. Black arrows and grey arrowheads indicate positions of anchor- and 3C- primers respectively. (B) Enlarged region encompassing the CTCF AD, the DMR0 and the IGF2 gene showing exons as boxes (blue for coding- and white for non-coding exons). Promoters are shown as arrows above the exons. The DMR0 region is shown as a yellow bar. (C) Enlargement of the intervening region between IGF2 and H19 showing the position of the CCD (red box) relative to the BglII and BamHI restriction sites. (D) Enlargement of ICR and H19 region. The CTCF/cohesin binding region is shown as red box and the position of the ICR is marked with a yellow box. (E) Enlargement of the enhancer and the CTCF DS region (green oval and red box respectively). (0.66 MB TIF) [file pgen.1000739.s001.tif]

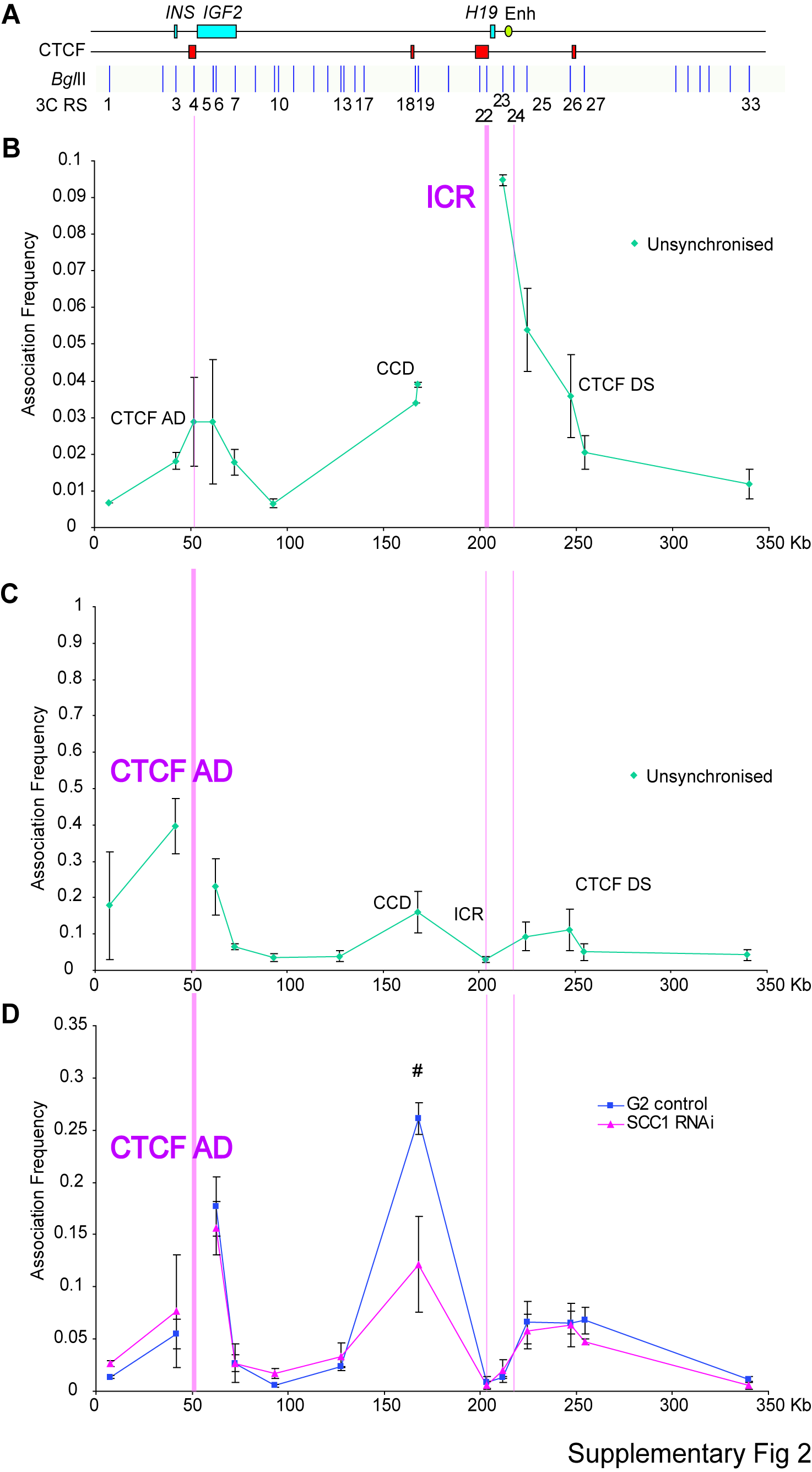

Supplement: Figure S2 — Chromatin conformation of the IGF2-H19 locus using BglII restriction. (A) Schematic representation of a 350 Kb genomic region including INS and the IGF2-H19 locus. Blue boxes represent the position of genes and the green oval depicts the localisation of the enhancer (Enh). Red boxes below represent the CTCF/cohesin binding regions CTCF AD, CCD, ICR, and CTCF DS. Positions of BglII restriction sites are marked with vertical blue lines and sites analysed by 3C are showed with numbers. Vertical pink lines in panel (B–D) indicate the position of the two different BglII restriction sites that were used as anchors. In each panel the respective anchor is marked with a thicker pink line. The X-axis is labelled according to the genomic position and position 0 was arbitrarily fixed 42 Kb upstream of INS. In (B,C) unsynchronised cells were analysed for the 3C interactions of the locus. (B) Associations detected with the anchor site in the ICR (restriction site 22 as anchor). The ICR associates with the CTCF AD and also with P2. (C) Associations with the CTCF AD (restriction site 4 as anchor). Interactions can be detected with the CCD and CTCF DS. (D) CTCF AD association frequencies (restriction site 4 as anchor) in cohesin depleted (SCC1 RNAi) and control (G2 control) cells synchronised in G2 phase. Interactions can be detected with the CCD and CTCF DS. Associations with the CCD were significantly decreased after SCC1 RNAi while interactions with the CTCF DS are only mildly affected. These results are similar to that obtained in Figure 7 with BamHI enzyme. (0.64 MB TIF) [file pgen.1000739.s002.tif]

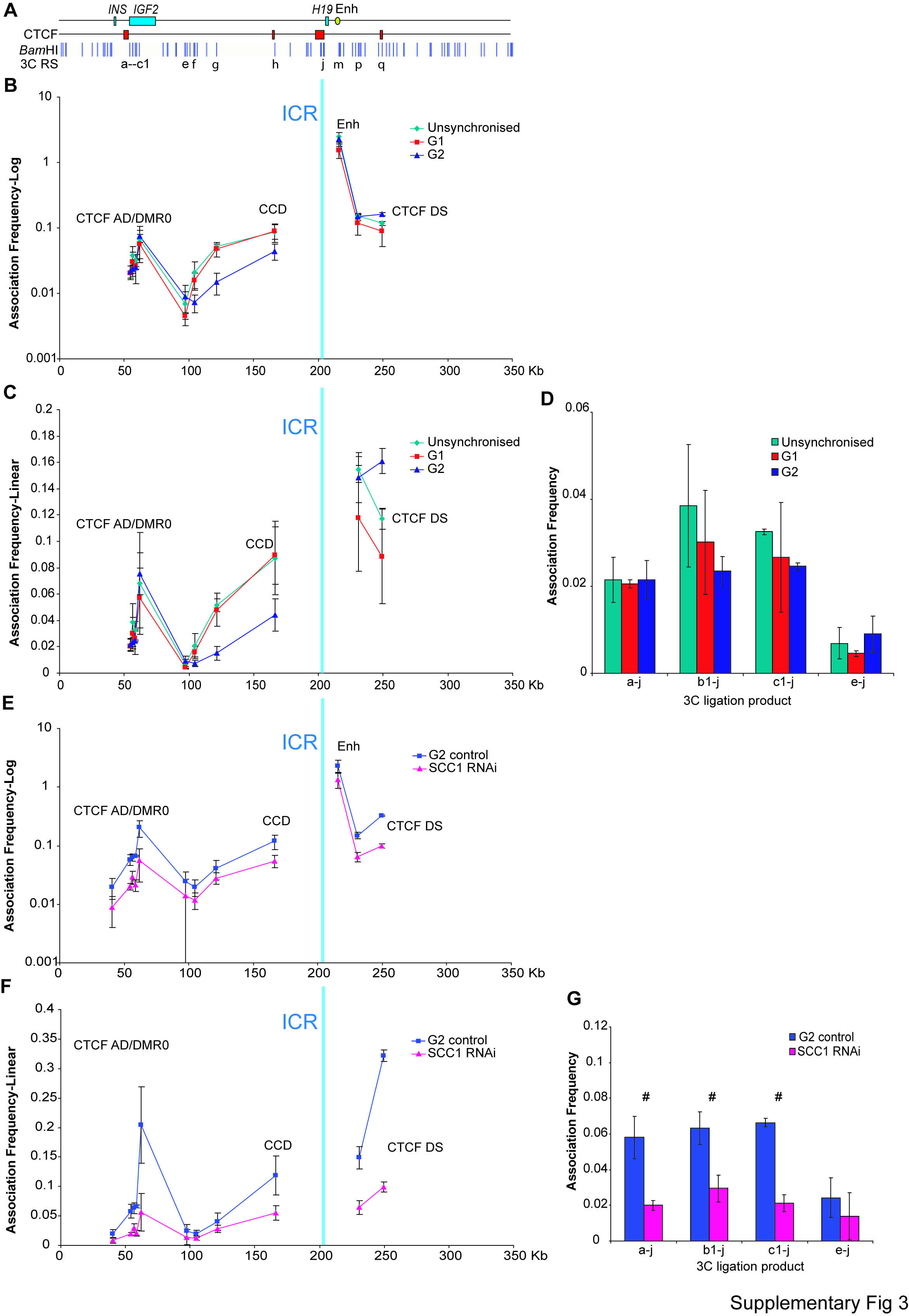

Supplement: Figure S3 — Chromatin conformation of the IGF2-H19 locus using an alternative anchor site in the ICR. (A) Schematic representation of a 350 Kb genomic region including INS and the IGF2/H19 locus. Blue boxes represent the position of genes, the green oval depicts the localisation of the enhancer (Enh) (see also Figure 1) and red boxes in the second line represent the CTCF/cohesin binding regions CTCF AD, CCD, ICR, and CTCF DS. Vertical blue lines in a row indicate the position of BamHI restriction sites within the locus and letters point out the restriction sites analysed by 3C (3C RS) in the graphs and histograms shown in the other panels. The blue vertical line across each graph indicates the position of the anchor restriction site (j) in the ICR. (B,C) Association frequencies of the ICR (anchor site j) throughout the locus are analysed in unsynchronised cells as well as in cells synchronised in G1 and G2 phases. Association frequencies are displayed on a log scale to include all data points (B) but also on a linear scale (C) to better visualise differences. For all three cell populations, associations of the ICR can be detected with the CTCF AD/DMR0 region as well as with the CTCF DS. The CCD is too close to the ICR to distinguish an interaction. ICR association frequencies with intervening regions between the CCD and the IGF2 gene (restriction sites e, f and g) vary between G1 and G2 phase. Since these are random ligation interactions we don't know what this means. We do not see this with an anchor near the k restriction site (Figure 3). The association frequencies between ICR and the CTCF AD/DMR0 were similar; however we do see a higher association frequency in G2 compared to G1 at the CTCF DS region. We also see this difference when we use the k primer as an anchor (Figure 3). The high interaction with the d restriction site is not reproducible. (D) Associations of the ICR with individual restriction sites close to the CTCF AD/DMR0 are depicted as histograms for higher res [file pgen.1000739.s003.tif]

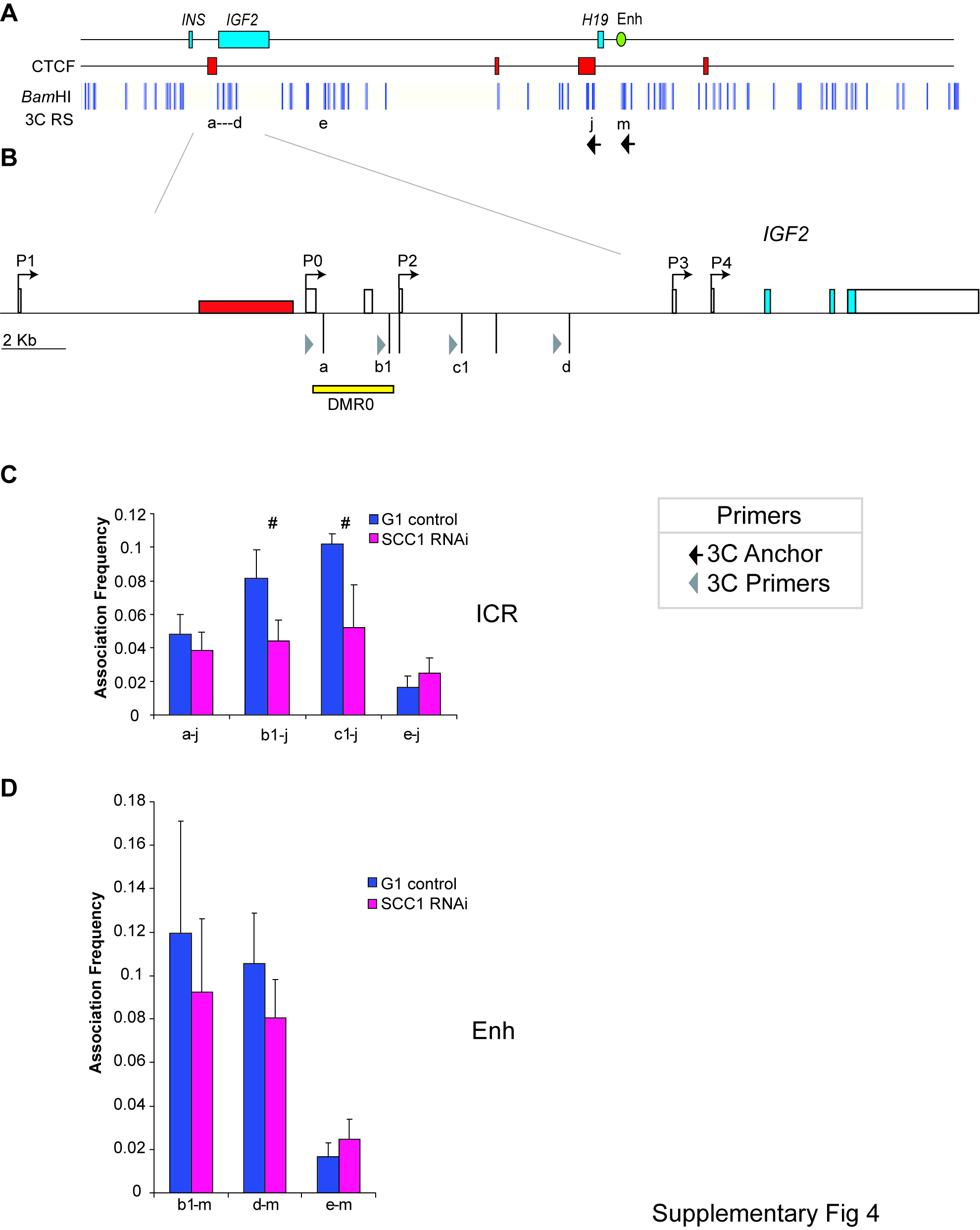

Supplement: Figure S4 — Effect of cohesin depletion on chromatin conformation in the IGF2/H19 locus in G1 phase cells. (A) Schematic representation of a 350 Kb genomic region including INS and the IGF2-H19 locus. Blue boxes represent the position of genes, the green oval depicts the localisation of the enhancer (Enh) (see also Figure 1) and red boxes in the second line represent the CTCF/cohesin binding regions CTCF AD, CCD, ICR, and CTCF DS. Vertical blue lines in a row indicate the position of BamHI restriction sites within the locus and letters point out the restriction sites analysed by 3C (3C RS). Anchor primers are shown as black arrows below the restriction site map. (B) Enlarged view of the region comprising the CTCF AD/DMR0 and the IGF2 gene showing exons as boxes (blue for coding and empty for non-coding exons). Promoters are shown as arrows above the exons and the DMR0 region is shown as a yellow bar. Reciprocal 3C primers are shown as grey arrowheads. (C) Association frequencies of the ICR (j-primer as anchor) with restriction sites in the CTCF AD/DMR0 (a and b1) and IGF2 gene promoter (c1) in cohesin depleted (SCC1 RNAi) and control (G1 control) cells synchronised in G1 phase. Association frequencies with restriction sites in IGF2 upstream region drop significantly in cohesin depleted cells compared to a random ligation with a restriction site in the intervening region (restriction site e) that is not reduced. (D) The effect of cohesin depletion on associations between the enhancer (m primer) and restriction sites close to promoters P2 (b1 restriction site), and P3 (d restriction site), was analysed. Upon cohesin depletion no significant change in the association frequencies was observed, confirming that these interactions occur independently of CTCF/cohesin binding (Table S1). # Denotes significant differences (P<0.05) between control and SCC1 RNAi. (0.74 MB TIF) [file pgen.1000739.s004.tif]

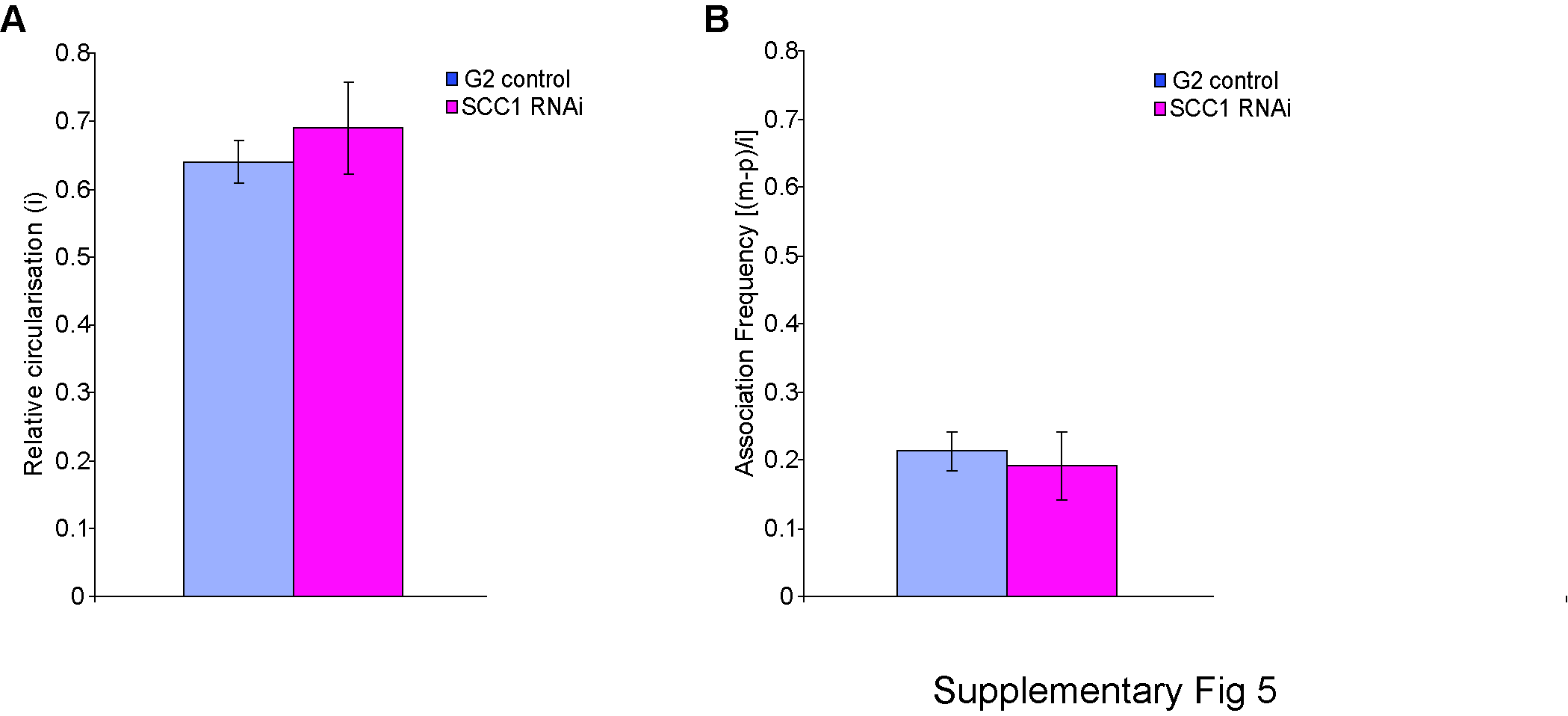

Supplement: Figure S5 — Effects of normalisation using circularisation of a restriction fragment as an internal control. BamHI restriction of i and j sites followed by random ligation results in circularisation of this fragment (i-fragment). Circularisation of i-fragment can be detected by using primers amplifying 180 bp spanning the re-ligated i and j sites. (Primer sequences are located in Table S3.) Inefficient digestion or ligation will give very low yields of this PCR product and therefore it is a good ligation and digestion control. (A) We found that the variation of i-fragment circularisation relative to the copy number of template between 3 biological replicates was low. There was also no significant difference in the relative circularisation frequency of i-fragment between control and cohesin depleted samples. (B) All interactions in our 3C samples were normalised using the circularisation frequency of the i-fragment. There was no significant difference between control and cohesin depleted samples at an interaction between the enhancer anchor (primer m) and a restriction site between the enhancer and the CTCF DS (site p). The interaction between m and p is not dependent on CTCF/cohesin and is therefore not expected to change after cohesin depletion. (0.29 MB TIF) [file pgen.1000739.s005.tif]
