# Supplementary material for: Cohesin Is Required for Higher-Order Chromatin Conformation at the Imprinted IGF2-H19 Locus
Source: PLoS Genet. 2009 Nov 26;5(11):e1000739. doi: 10.1371/journal.pgen.1000739 (PMC2776306; doi:10.1371/journal.pgen.1000739)
Supplement: Table S1 — Statistical analysis of difference in locus-wide association frequencies with ICR, enhancer, CTCF AD/DMR0, and CTCF DS anchors. (0.31 MB DOC) [file pgen.1000739.s006.doc]

**Table S1: Statistical Analysis of Difference in Locus-Wide Association Frequencies with ICR, Enhancer, CTCF AD/DMR0 and CTCF DS anchors.**

| **G2 : ICR anchor interactions (k restriction site)** | | | | | | | | |  | |  |  |  |  |
| --- | --- | --- | --- | --- | --- | --- | --- | --- | --- | --- | --- | --- | --- | --- |
| **RS** |  |  | **Position** | **Control** | | **SSC1 RNAi** | |  | | | | | | |
|  |  |  |  | **Summary Statistics** | | | | | | | | | | |
|  |  |  |  | Mean (SD) | | Mean (SD) | | Difference | 95% CI. | P value | Summary | | | |
| z |  |  | 40.302 | 0.061 | (0.01) | 0.030 | (0.01) | -0.032 | -0.1094 to 0.04626 | P > 0.05 | ns | | | |
| a |  |  | 54.393 | 0.227 | (0.07) | 0.109 | (0.03) | -0.118 | -0.1958 to -0.04013 | P<0.001 | *** | | | |
| b1 |  |  | 56.456 | 0.051 | (0.03) | 0.0409 | (0.01) | -0.011 | -0.08881 to 0.06685 | P > 0.05 | ns | | | |
| c1 |  |  | 58.6 | 0.074 | (0.01) | 0.062 | (0.01) | -0.013 | -0.09050 to 0.06517 | P > 0.05 | ns | | | |
| d |  |  | 61.818 | 0.079 | (0.01) | 0.05 | (0.02) | -0.029 | -0.1064 to 0.04931 | P > 0.05 | ns | | | |
| e1 |  |  | 100.847 | 0.007 | (0.01) | 0.011 | (0.01) | 0.004 | -0.07382 to 0.08184 | P > 0.05 | ns | | | |
| f |  |  | 104.491 | 0.025 | (0.01) | 0.015 | (0.00) | -0.011 | -0.08862 to 0.06704 | P > 0.05 | ns | | | |
| h |  |  | 166.305 | 0.284 | (0.02) | 0.213 | (0.01) | -0.071 | -0.1492 to 0.006462 | P > 0.05 | ns | | | |
| h1 |  |  | 191.916 | 0.493 | (0.05) | 0.34 | (0.12) | -0.153 | -0.2308 to -0.07518 | P<0.001 | *** | | | |
| m |  |  | 215.691 | 0.804 | (0.24) | 0.51 | (0.08) | -0.294 | -0.3716 to -0.2160 | P<0.001 | *** | | | |
| p |  |  | 230.72 | 0.427 | (0.10) | 0.23 | (0.08) | -0.128 | -0.2056 to -0.04990 | P<0.001 | *** | | | |
| q |  |  | 249.286 | 0.440 | (0.05) | 0.155 | (0.03) | -0.286 | -0.3634 to -0.2077 | P<0.001 | *** | | | |
| r |  |  | 257.824 | 0.242 | (0.09) | 0.102 | (0.02) | -0.140 | -0.2177 to -0.06200 | P<0.001 | *** | | | |
| Data shown graphically in Figs. 7B and C | | | | | | | | | | | | | | |
| **G2: ICR anchor interactions (j restriction site)** | | | | | | | | | | | | | | |
| z |  |  | 40.302 | 0.020 | (0.01) | 0.0088 | (0.00) | -0.011 | -0.19 to 0.16 | P > 0.05 | ns | | | |
| a |  |  | 54.393 | 0.058 | (0.01) | 0.020 | (0.00) | -0.038 | -0.21 to 0.14 | P< 0.05 | * | | | |
| b1 |  |  | 56.456 | 0.061 | (0.01) | 0.030 | (0.01) | -0.031 | -0.35 to -0.0050 | P < 0.05 | * | | | |
| c1 |  |  | 58.6 | 0.066 | (0.00) | 0.021 | (0.00) | -0.045 | -0.22 to 0.13 | P < 0.05 | * | | | |
| d |  |  | 61.818 | 0.20 | (0.06) | 0.056 | (0.03) | -0.15 | -0.32 to 0.028 | P > 0.05 | ns | | | |
| e |  |  | 97.284 | 0.024 | (0.01) | 0.014 | (0.01) | -0.010 | -0.18 to 0.16 | P > 0.05 | ns | | | |
| f |  |  | 104.491 | 0.020 | (0.01) | 0.012 | (0.00) | -0.0078 | -0.18 to 0.17 | P > 0.05 | ns | | | |
| g |  |  | 121.37 | 0.041 | (0.01) | 0.028 | (0.01) | -0.013 | -0.19 to 0.16 | P > 0.05 | ns | | | |
| h |  |  | 166.305 | 0.12 | (0.03) | 0.055 | (0.01) | -0.064 | -0.24 to 0.11 | P > 0.05 | ns | | | |
| p |  |  | 230.72 | 0.15 | (0.02) | 0.064 | (0.01) | -0.084 | -0.26 to 0.090 | P<0.001 | ns | | | |
| q |  |  | 249.286 | 0.32 | (0.01) | 0.099 | (0.01) | -0.22 | -0.40 to -0.049 | P<0.001 | ** | | | |
| Data shown graphically in Fig. S3E-G. | | | | | | | | | | | | | | |
| **G1: ICR anchor interactions (j restriction site)** | | | | | | | | | | | | | | |
| a |  |  | 54.393 | 0.058 |  | 0.041 | (0.00) | -0.018 |  |  |  | | | |
| b1 |  |  | 56.456 | 0.077 | (0.01) | 0.041 | (0.01) | -0.035 | -0.37 to 0.10 | P < 0.05 | * | | | |
| c1 |  |  | 58.6 | 0.11 | (0.04) | 0.053 | (0.02) | -0.055 | -0.39 to 0.11 | P< 0.05 | * | | | |
| d |  |  | 61.818 | 0.10 | (0.03) | 0.053 |  | -0.049 |  |  |  | | | |
| e |  |  | 97.284 | 0.014 | (0.00) | 0.020 | (0.00) | 0.0063 | -0.33 to 0.34 | P > 0.05 | ns | | | |
| f |  |  | 104.491 | 0.031 | (0.02) | 0.038 | (0.01) | 0.0064 | -0.33 to 0.35 | P > 0.05 | ns | | | |
| g |  |  | 121.38 | 0.056 | (0.00) | 0.039 | (0.04) | -0.017 | -0.36 to 0.32 | P > 0.05 | ns | | | |
| p |  |  | 215.691 | 0.28 | (0.14) | 0.17 | (0.06) | -0.11 | -0.45 to 0.23 | P > 0.05 | ns | | | |
| Data shown graphically in Fig S4C | | | | | | | | | | | | | | |
| **G2: Enh anchor interactions (m restriction site)** | | | | | | | | | | | | | | |
| z |  |  | 40.302 | 0.0076 | (0.01) | 0.0090 | (0.01) | 0.0014 | -0.099 to 0.10 | P > 0.05 | ns | | | |
| a |  |  | 54.393 | 0.039 | (0.04) | 0.040 | (0.00) | 0.00063 | -0.10 to 0.10 | P > 0.05 | ns | | | |
| b2 |  |  | 56.697 | 0.054 | (0.05) | 0.045 | (0.01) | -0.0087 | -0.11 to 0.091 | P > 0.05 | ns | | | |
| c1 |  |  | 58.6 | 0.022 | (0.02) | 0.035 | (0.01) | 0.013 | -0.087 to 0.11 | P > 0.05 | ns | | | |
| c2 |  |  | 59.624 | 0.013 | (0.01) | 0.011 |  | -0.0017 | -0.10 to 0.099 | P > 0.05 | ns | | | |
| d |  |  | 61.818 | 0.012 | (0.01) | 0.0096 | (0.01) | -0.0029 | -0.10 to 0.097 | P > 0.05 | ns | | | |
| e |  |  | 97.284 | 0.0080 | (0.01) | 0.0052 |  | -0.0029 | -0.10 to 0.097 | P > 0.05 | ns | | | |
| f |  |  | 104.491 | 0.0089 | (0.01) | 0.0051 | (0.00) | -0.0038 | -0.10 to 0.096 | P > 0.05 | ns | | | |
| h |  |  | 166.305 | 0.040 | (0.04) | 0.025 | (0.01) | -0.015 | -0.12 to 0.085 | P > 0.05 | ns | | | |
| i |  |  | 202.134 | 0.055 | (0.05) | 0.070 |  | 0.015 | -0.086 to 0.11 | P > 0.05 | ns | | | |
| p |  |  | 230.72 | 0.056 | (0.06) | 0.051 | (0.00) | -0.0052 | -0.11 to 0.095 | P > 0.05 | ns | | | |
| q |  |  | 249.286 | 0.025 | (0.03) | 0.023 | (0.01) | -0.0027 | -0.10 to 0.097 | P > 0.05 | ns | | | |
| r |  |  | 257.824 | 0.015 | (0.02) | 0.012 |  | -0.0038 | -0.10 to 0.096 | P > 0.05 | ns | | | |
| t |  |  | 337.493 | 0.014 | (0.01) | 0.0070 |  | -0.0067 | -0.11 to 0.093 | P > 0.05 | ns | | | |
| Data shown graphically in Fig 7D and E | | | | | | | | | | | | | | |
| a |  |  | 54.393 | 0.22 | (0.05) | 0.080 | (0.02) | -0.14 | -0.24 to -0.041 | P<0.001 | *** | | | |
| b1 |  |  | 56.456 | 0.16 | (0.05) | 0.10 | (0.02) | -0.060 | -0.16 to 0.040 | P > 0.05 | ns | | | |
| c1 |  |  | 58.6 | 0.14 | (0.03) | 0.062 | (0.02) | -0.074 | -0.17 to 0.027 | P > 0.05 | ns | | | |
| d |  |  | 61.818 | 0.12 | (0.04) | 0.055 | (0.02) | -0.064 | -0.16 to 0.036 | P > 0.05 | ns | | | |
| Data not shown graphically | | | | | | | | | | | | | | |
| **G1 Enh anchor interactions (m restriction site)** | | | | | | | | | | | | | | |
| b1 |  |  | 56.456 | 0.12 | (0.07) | 0.096 | (0.03) | -0.028 | -0.13 to 0.072 | P > 0.05 | ns | | | |
| c1 |  |  | 58.6 | 0.12 | (0.00) | 0.13 | (0.03) | 0.011 | -0.089 to 0.11 | P > 0.05 | ns | | | |
| d |  |  | 61.818 | 0.15 | (0.02) | 0.095 | (0.00) | -0.058 | -0.16 to 0.042 | P > 0.05 | ns | | | |
| e |  |  | 97.284 | 0.070 | (0.04) | 0.052 | (0.01) | -0.018 | -0.12 to 0.082 | P<0.01 | ** | | | |
| f |  |  | 104.491 | 0.13 | (0.03) | 0.098 | (0.04) | -0.031 | -0.13 to 0.069 | P > 0.05 | ns | | | |
| h |  |  | 166.305 | 0.54 | (0.06) | 0.39 | (0.06) | -0.15 | -0.25 to -0.054 | P > 0.05 | ns | | | |
| p |  |  | 230.72 | 0.71 | (0.11) | 0.52 | (0.13) | -0.19 | -0.29 to -0.086 | P > 0.05 | ns | | | |
| q |  |  | 249.286 | 0.50 | (0.02) | 0.26 | (0.12) | -0.24 | -0.34 to -0.14 | P > 0.05 | ns | | | |
| Data shown graphically in Fig S4D | | | | | | | | | | | | | | |
| **G2 CTCF AD/DMR0 interactions (b1 restriction site)** | | | | | | | | | | | | | | |
| z |  |  | 40.302 | 0.0067 | (0.00) | 0.0093 | (0.00) | 0.0026 | -0.26 to 0.26 | P > 0.05 | ns | | | |
| a |  |  | 54.393 | 0.53 | (0.11) | 0.55 | (0.02) | 0.017 | -0.24 to 0.28 | P > 0.05 | ns | | | |
| c1 |  |  | 58.6 | 0.43 | (0.08) | 0.28 | (0.06) | -0.15 | -0.41 to 0.11 | P > 0.05 | ns | | | |
| c2 |  |  | 59.624 | 0.24 | (0.05) | 0.22 | (0.02) | -0.019 | -0.28 to 0.24 | P > 0.05 | ns | | | |
| d |  |  | 61.818 | 0.046 | (0.01) | 0.044 | (0.00) | -0.0016 | -0.26 to 0.26 | P > 0.05 | ns | | | |
| e |  |  | 97.284 | 0.028 | (0.01) | 0.018 | (0.00) | -0.0095 | -0.27 to 0.25 | P > 0.05 | ns | | | |
| e1 |  |  | 100.847 | 0.011 | (0.00) | 0.012 |  | 0.0013 | -0.26 to 0.26 | P > 0.05 | ns | | | |
| f |  |  | 104.491 | 0.016 | (0.00) | 0.0088 | (0.00) | -0.0069 | -0.27 to 0.25 | P > 0.05 | ns | | | |
| g |  |  | 121.37 | 0.022 | (0.01) | 0.016 | (0.00) | -0.0066 | -0.27 to 0.25 | P > 0.05 | ns | | | |
| h |  |  | 166.305 | 0.50 | (0.03) | 0.35 | (0.09) | -0.15 | -0.41 to 0.11 | P< 0.05 | ** | | | |
| h1 |  |  | 191.916 | 0.079 | (0.03) | 0.052 | (0.01) | -0.027 | -0.29 to 0.23 | P > 0.05 | ns | | | |
| k |  |  | 204.183 | 0.020 | (0.00) | 0.015 | (0.00) | -0.0052 | -0.26 to 0.25 | P > 0.05 | ns | | | |
| m |  |  | 215.691 | 0.013 | (0.01) | 0.0077 | (0.00) | -0.0058 | -0.27 to 0.25 | P > 0.05 | ns | | | |
| p |  |  | 230.72 | 0.012 | (0.00) | 0.0080 | (0.00) | -0.0035 | -0.26 to 0.26 | P > 0.05 | ns | | | |
| q |  |  | 249.286 | 0.11 | (0.02) | 0.084 | (0.01) | -0.023 | -0.28 to 0.24 | P > 0.05 | ns | | | |
| Data shown graphically in Fig 7G and H | | | | | | | | | | | | | | |
| h |  |  | 166.305 | 2.0 | (0.10) | 0.64 | (0.18) | 1.370 | 1.1 to 1.6 | P<0.001 | *** | | | |
| j |  |  | 203.957 | 0.063 | (0.01) | 0.024 | (0.02) | 0.03908 | -0.22 to 0.30 | P > 0.05 | ns | | | |
| m |  |  | 215.691 | 0.22 | (0.02) | 0.094 | (0.12) | 0.1255 | -0.13 to 0.39 | P > 0.05 | ns | | | |
| Data not shown graphically | | | | | | | | | | | | | | |
| ***Bgl*2 G2 – CTCF AD anchor interactions (restriction site no 4)** | | | | | | | | | | | | | | |
| 1 |  |  | 7 | 0.014 | (0.00) | 0.028 | (0.00) | 0.014 | -0.026 to 0.054 | P > 0.05 | ns | | | |
| 3 |  |  | 42 | 0.055 | (0.01) | 0.077 | (0.05) | 0.022 | -0.018 to 0.062 | P > 0.05 | ns | | | |
| 6 |  |  | 63 | 0.18 | (0.03) | 0.16 | (0.03) | -0.021 | -0.061 to 0.019 | P > 0.05 | ns | | | |
| 7 |  |  | 72 | 0.027 | (0.01) | 0.027 | (0.02) | -0.00 | -0.040 to 0.040 | P > 0.05 | ns | | | |
| 10 |  |  | 93 | 0.0052 | (0.00) | 0.017 | (0.00) | 0.012 | -0.029 to 0.052 | P > 0.05 | ns | | | |
| 13 |  |  | 127 | 0.023 | (0.00) | 0.033 | (0.01) | 0.010 | -0.030 to 0.050 | P > 0.05 | ns | | | |
| 19 |  |  | 168 | 0.26 | (0.01) | 0.12 | (0.05) | -0.14 | -0.18 to -0.099 | P<0.001 | *** | | | |
| 22 |  |  | 204 | 0.0086 | (0.01) | 0.0038 |  | -0.0048 | -0.045 to 0.036 | P > 0.05 | ns | | | |
| 23 |  |  | 212 | 0.013 | (0.00) | 0.020 | (0.01) | 0.0064 | -0.034 to 0.047 | P > 0.05 | ns | | | |
| 24 |  |  | 224 | 0.066 | (0.02) | 0.057 | (0.02) | -0.0085 | -0.049 to 0.032 | P > 0.05 | ns | | | |
| 26 |  |  | 247 | 0.065 | (0.01) | 0.064 | (0.02) | -0.0017 | -0.042 to 0.039 | P > 0.05 | ns | | | |
| 27 |  |  | 254 | 0.068 | (0.01) | 0.047 | (0.00) | -0.021 | -0.061 to 0.020 | P > 0.05 | ns | | | |
| 33 |  |  | 340 | 0.011 | (0.00) | 0.0037 |  | -0.0075 | -0.048 to 0.033 | P > 0.05 | ns | | | |
| Data shown graphically in Fig S2E  Bonferrroni post test following two-way Anova analysis with Bonferroni post-test (GraphPad Prism version 5.01 for Windows www.graphpad.com. Treatment (RNAi or control) the first factor, interacting restriction sites the second factor. The Bonferroni correction lowers the P value considered to be significant to 0.05 divided by the number of comparisons. (Thus in n rows of data with two columns (Control and RNAi), the P value has to be less than 0.05/n, for any particular row in order to be considered significant with P<0.05). This correction ensures that the 5% probability applies to the entire family of comparisons, and not separately to each individual comparison.  Where SD is missing, 3 data points for biological replicates were not available for analysis. Four different sets of experiments were done and data are colour coded to identify the experiment: Grey cells in table represent a complete data set of 3 biological replicate control and RNAi samples in G2 phase processed for 3C simultaneously and analysed for all anchor primers; Pink cells represent data on a limited number of interactions from 3 biological replicate control and RNAi samples | | | | | | | | | | | | | | |
