# Supplementary material for: Cohesin Is Required for Higher-Order Chromatin Conformation at the Imprinted IGF2-H19 Locus
Source: PLoS Genet. 2009 Nov 26;5(11):e1000739. doi: 10.1371/journal.pgen.1000739 (PMC2776306; doi:10.1371/journal.pgen.1000739)
Supplement: Table S2 — ChIP primers. (0.04 MB DOC) [file pgen.1000739.s007.doc]

**Table S2: ChIP primers**

|  |  | 5'-------------------------------------------------3' |
| --- | --- | --- |
| CTCF AD 1 | FW | CTCTTCTCTCAATTCCCAAGGTTT |
|  | REV | CACCTTTCTGAAGCATCCGTTT |
| CTCF AD 2 | FW | ACCCATGGCACCCTGACA |
|  | REV | ACCAAGCTTTCGCCATTTAGC |
| DMR0 a | FW | ATCATCGTCCAGGCAGTTTC |
|  | REV | TTCCTTGCAAAAGCCTCAGT |
| DMR0 b | FW | GAACCTAAAAACCCAAGGAAAGC |
|  | REV | AGCCACCTTTCACCCCTCTT |
| P2 | FW | CATATCCCCCACGTACTTTTGG |
|  | REV | ATTTTACAAACCCAGCTCCTTTCTC |
| P3 | FW | GCTGACCTCATTTCCCGATA |
|  | REV | GTGTCGCAAACCGAACAG |
| P4 | FW | TCCTGTGAAAGAGACTTCCAGCTT |
|  | REV | ACTCCACAGCCCTGGTTACCT |
| Exon 9 | FW | CCACGATGCTCCCCATACC |
|  | REV | CCGCCAGACTTCCCACACT |
| CCD | FW | GGAGGAGGACAGAGGCAAGAG |
|  | REV | AACAAAATTTCAGCCGGTTCA |
| ICR | FW | TGAATTTGCCCACAGGTGTTC |
|  | REV | GCCTTGGGTCACCTTCAGACT |
| Enh | FW | CTGGGCAAGAGAGTGACACC |
|  | REV | GTGCCAATTCCTCAAGGCTA |
| CTCF DS | FW | TTTCTTTGGGCTCTTCATTTTGA |
|  | REV | CCCCCCAGGATACGTAAGAAA |
