# Supplementary material for: Cohesin Is Required for Higher-Order Chromatin Conformation at the Imprinted IGF2-H19 Locus
Source: PLoS Genet. 2009 Nov 26;5(11):e1000739. doi: 10.1371/journal.pgen.1000739 (PMC2776306; doi:10.1371/journal.pgen.1000739)
Supplement: Table S3 — 3C primers for BamH1 template. (0.12 MB DOC) [file pgen.1000739.s008.doc]

**Table S3: 3C Primers for *Bam*H1 template**

| **Anchor Primers** | | **Reciprocal Primer** | |
| --- | --- | --- | --- |
|  | |
| I**CR Anchor** | | | |
|  | | | |
| k | FW: TGAATTTGCCCACAGGTGTTC **†** |  |  |
|  |  | z | FW: GGCTGGATTTCAGACTCTGTAAGAT |
|  |  | a | FW: CAGGCAGTTTCGGCAGAGA |
|  |  | a | REV**:** TTCCTTGCAAAAGCCTCAGT**†** |
|  |  | b1 | FW: GAACCTAAAAACCCAAGGAAAGC**†** |
|  |  | c1 | REV: CCTTGGCGTTGGGCAAT |
|  |  | c2 | FW: GAGCTCAACAATTAGCCCTTGATC |
|  |  | d | FW: CTGAAGCCACAGTAATTATGAAGGTT |
|  |  | e1 | FW: TGAGAAACACACCTGAGAAACCAA |
|  |  | f | FW: AAGGGGCATTCAGGGATAAG |
|  |  | h | FW: GGAGGAGGACAGAGGCAAGAG**†** |
|  |  | h1 | FW: GGAGGAGGACAGAGGCAAGAG |
|  |  | m | FW: GTCACTCATGCACGCCAATG |
|  |  | p | FW: CCCCAACTTGCCTGGACTTT |
|  |  | q | FW: GTTGCCCTGCCCTGTCTCT |
|  |  | r | FW: TGAGCTAGGAGCTGAGGTCCTT |
|  |  | s | REV: AGAGCAAATCTCACTGCCTTTTTT |
| j | REV: CTGCACCCACGATAATGGATT | a | FW: As above |
|  |  | b1 | FW: As above |
|  |  | c1 | FW: TTGCCAAGCTAATGTGAAAGAGAT |
|  |  | d | FW: As above |
|  |  | e | FW: GGAGTGGTGGATAGGGAGGAA |
|  |  | f | FW: As above |
|  |  | g | FW:CAAATACCGCATGTTCTCACTTATAAG |
|  |  | h | FW: As above |
| j | FW: CCTAAGTGGCCAGACATTAACATTC | m | REV: GGTCCGAAATTCCATGTCCTT |
|  |  | p | REV: TGTGGGTGGCCGCATT |
|  |  | q | REV: CCCCACCTCCCATGAAAACT |
|  | | | |
| **CTCF AD/DMR0 Anchor** | | | |
|  | | | |
| b1 | FW: GAACCTAAAAACCCAAGGAAAGC**†** |  |  |
|  |  | z | FW: As above |
|  |  | a | FW: As above |
|  |  | c1 | FW: As above |
|  |  | c2 | FW: As above |
|  |  | d | FW: As above |
|  |  | e | FW: As above |
|  |  | e1 | REV: TGAACAGTTGGATGGAGGAATG |
|  |  | f | FW: As above |
|  |  | g | FW: As above |
|  |  | h | FW: As above |
|  |  | h1 | FW: TGGGAGGGTGGTCTGAGTATG |
|  |  | j | FW: CCTAAGTGGCCAGACATTAACATTC |
|  |  | m | FW: As above |
|  |  | p | FW: As above |
|  |  | q | FW: As above |
|  |  | r | FW: As above |
|  |  | t | FW: As above |
|  | | | |
| **Enhancer Anchor** | | | |
|  | | | |
| m | REV: CCTTCCTGCTGCTCAGAGGTT |  |  |
|  |  | z | FW: As above |
|  |  | a | FW: As above |
|  |  | b1 | FW: As above |
|  |  | b2 | REV: |
|  |  | c1 | FW: As above |
|  |  | c2 | FW: As above |
|  |  | d | FW: As above |
|  |  | e | FW: GGAGTGGTGGATAGGGAGGAA |
|  |  | e1 | REV: TGAACAGTTGGATGGAGGAATG |
|  |  | f | FW: As above |
|  |  | h | REV: AACAAAATTTCAGCCGGTTCA |
|  |  | p | FW: As above |
|  |  | q | FW: As above |
|  |  | r | FW: As above |
|  |  | t | FW: As above |
| m | FW: CCTTCCTGCTGCTCAGAGGTT | a | REV As above |
|  |  | c2 | REV: AGCCTGGGAGAAAGCACATCT**†** |
|  | | | |
| **CTCF DS Anchor**: | | | |
|  | | | |
| q | FWD: GTTGCCCTGCCCTGTCTCT |  |  |
|  |  | z | FW: As above |
|  |  | a | REV As above |
|  |  | b1 | FW: As above |
|  |  | c1 | FW: As above |
|  |  | d | FW: As above |
|  |  | e | REV: GCACACACCAGGGTGCAA |
|  |  | e1 | REV: As above |
|  |  | h | REV: As above |
|  |  | j | FW: As above |
|  |  | m | FW: As above |
|  |  | p | FW: As above |
|  |  | q | FW: As above |
|  |  | r | FW: As above |
| **Normalisation Primers** | |  |  |
| i | FW: ATTCTCATTCAATGCAGGTTTGAG | |  |
| i | REV:  TGTTTCCATACCTTCAACTGATTCC | |  |
|  |  |  |  |

FW= forward primer

REV= reverse primer

**†** = SNP amplifying primer
