# Supplementary material for: Cohesin Is Required for Higher-Order Chromatin Conformation at the Imprinted IGF2-H19 Locus
Source: PLoS Genet. 2009 Nov 26;5(11):e1000739. doi: 10.1371/journal.pgen.1000739 (PMC2776306; doi:10.1371/journal.pgen.1000739)
Supplement: Table S4 — 3C primers for BglII template. (0.06 MB DOC) [file pgen.1000739.s009.doc]

**Table S4: 3C Primers for *Bgl*II template**

| **Anchor Primers** | | **Reciprocal Primer** | |
| --- | --- | --- | --- |
|  | |
| I**CR Anchor** | | | |
| 22 | REV: TTTTGGTGGAACACACTGTGATC |  |  |
|  |  | 1 | REV: CGGAAGCCTGTGGACAGTG |
|  |  | 3 | REV: CCCGCTGGCTTTATAGTCTCA |
|  |  | 4 | FW: GAAGCGAGTTAAGAACGAAATTCAG |
|  |  | 5 | FW: CCTCCAAGGACTCTGAGCAACT |
|  |  | 7 | REV: CCGCCAGACTTCCCACACT |
|  |  | 10 | FW: TGCCTTTTCGAGGACGTCAT |
|  |  | 18 | FW: CCGCCACCCGCAAAG |
|  |  | 19 | REV: CAGGCAAGGGAAAGGAGAGAC |
|  |  | 23 | REV: GGGGGAGAAAGAGAAAGGAG |
|  |  | 25 | REV: GGTGCCAGATAGCTGCATGTC |
|  |  | 26 | REV: AGGTACCTCTGAGCAGTTGAATCC |
|  |  | 27 | REV: GTGGAGGTGCTTGTGAAATCC |
|  |  | 33 | REV: CCCTTGCTTCCCTCAGTGTTC |
|  |  |  |  |
| **CTCF AD Anchor** | |  |  |
| 4 | FW: GAAGCGAGTTAAGAACGAAATTCAG | 1 | REV As above |
|  |  | 3 | REV As above |
|  |  | 4 | FW As above |
|  |  | 6 | FW: GCCACTTTCGTTTGCTCATT |
|  |  | 7 | REV As above |
|  |  | 10 | FW As above |
|  |  | 13 | FW: CTCATCTCACCCAGAATCAACCT |
|  |  | 19 | REV As above |
|  |  | 22 | REV TTTTGGTGGAACACACTGTGATC |
|  |  | 23 | REV As above |
|  |  | 24 | REV: GTGCCAATTCCTCAAGGCTA |
|  |  | 26 | REV As above |
|  |  | 27 | REV As above |
|  |  | 33 | REV As above |

FW= forward primer

REV= reverse primer
